# Characterization of OsCAF1 Protein Function in Rice Response to Thermal Stress

**DOI:** 10.3390/plants14071036

**Published:** 2025-03-27

**Authors:** Vu-Bao Nguyen, Chung-An Lu

**Affiliations:** Department of Life Sciences, National Central University, 300 Jhong-Da Road, Jhong-Li District, Taoyuan City 320, Taiwan

**Keywords:** heat stress, processing bodies (PBs), CCR4-associated factor 1 (CAF1), eukaryotic initiation factor 4AII (eIF4AII), DEAD-box ATP-dependent RNA helicase 8 (RH8), rice (*Oryza sativa*)

## Abstract

Heat stress is a critical environmental challenge that disrupts rice growth, development, and productivity and poses a significant threat to global food security. The CCR4-NOT protein complex, particularly its CCR4-associated factor 1 (CAF1) subunit, plays a crucial role in the dynamic regulation of gene expression by mediating mRNA de-adenylation, a key step in mRNA degradation and turnover. However, the specific function of OsCAF1 proteins under heat stress in rice remains poorly understood. In this study, we investigated the dynamic subcellular localization of OsCAF1A in response to elevated temperatures and its role in heat stress tolerance. Under normal conditions, OsCAF1A is diffusely localized to the cytoplasm. However, OsCAF1A predominantly localizes to processing bodies (PBs) under heat stress. The results of interaction studies revealed that two DEAD-box RNA helicases, OseIF4AIIb and OsRH8, modulate the re-localization of OsCAF1A, by OseIF4AIIb inhibiting and OsRH8 promoting its association with PBs during heat stress. Furthermore, *OsCAF1A* mRNA was more abundantly expressed in rice seedlings than other *OsCAF1* genes and is further upregulated by high temperature. The overexpression of *OsCAF1A* significantly enhanced heat tolerance, whereas mutants exhibited increased heat sensitivity. These findings underscore the potential of OsCAF1A as a tool to improve crop resilience to climate change.

## 1. Introduction

Global warming has significantly increased the frequency and severity of heat extremes, posing a profound threat to agricultural productivity by adversely affecting plant growth, development, and reproduction [1,2]. Rice (*Oryza sativa*) is a staple food crop essential for global security, sustaining nearly half the world’s population (http://www.fao.org/faostat/zh/#data, accessed on 4 November 2024). However, heat stress severely affects rice growth and development, reducing seedling vigor, inhibiting pollen viability and germination, and causing spikelet sterility during the reproductive stage, ultimately leading to significant yield losses [3,4,5,6,7,8]. Therefore, understanding the molecular mechanisms that regulate plant heat stress responses is crucial for developing climate-resilient rice and other crops.

In plants, including rice, heat stress triggers an intricate network of signaling pathways and regulatory mechanisms that orchestrate adaptive responses to mitigate the detrimental effects of elevated temperatures. In previous studies, the primary focus has been heat shock transcription factors (HSFs) [9], and various transcription factor families, including NAC, WRKY, basic leucine zipper (bZIP), and MYB [10,11,12,13]. Additionally, the regulation of mRNA translation, decay, and sequestration dynamically fine-tunes gene expression under biotic and abiotic stress conditions, thereby supporting proper plant growth and development [14,15,16,17].

The Carbon Catabolite Repression 4-Negative On TATA-less (CCR4-NOT) complex is a key player in mRNA regulation, which plays a crucial role in various facets of eukaryotic gene expression, particularly in translational repression and mRNA de-adenylation [18]. Among the subunits of CCR4-NOT, CCR4-associated factor 1 (CAF1, also known as POP2 or CNOT7) is crucial for cytoplasmic de-adenylation, facilitating mRNA decay [19,20,21]. In contrast to yeasts and animals, which have limited *CAF1* genes, higher plants like *Arabidopsis* and rice have multiple CAF1 family members [22,23]. These plant CAF1 proteins participate in diverse developmental processes and stress responses [15,16,24,25].

Moreover, mRNAs can be dynamically sequestered into cytoplasmic structures, including processing bodies (PBs) and stress granules (SGs), which play a pivotal role in orchestrating cellular stress responses [26,27]. These dynamic structures play a central role in regulating mRNA stability and decay, with PBs acting as key sites for mRNA decay to maintain gene expression homeostasis under stress conditions. The assembly and function of PBs are tightly linked to the activity of the CCR4-NOT complex, particularly its catalytic subunit, CCR4-associated factor 1 (CAF1) [28,29].

Emerging evidence suggests that DEAD-box RNA helicases modulate mRNA sequestration and degradation by interacting with the CCR4-NOT complex, thereby influencing the dynamics of PBs and SGs in response to stress. DEAD-box RNA helicases are present in all eukaryotes and most prokaryotes, and contain a conserved helicase core domain that is vital for RNA metabolism and plays key roles in plant growth, development, and stress tolerance [30,31]. In human cells, two DEAD-box helicases, eIF4A2 and DDX6, function as key effectors of the CCR4-NOT complex: DDX6 enhances the activity of the human CAF1 homolog CNOT7 to promote mRNA decay, whereas eIF4A2 suppresses this activity [32,33]. However, the role of these helicases in CAF1 regulation in plant systems, especially under heat stress, remains largely unexplored.

Although CAF1 proteins play a common role in cytoplasmic mRNA de-adenylation across eukaryotes, their subcellular localization remains largely uncharacterized. For example, Pop2p remains in the cytoplasm under normal conditions, but relocates to PBs in response to stress, a process mediated by the DEAD-box RNA helicase Dhh1p [34,35]. In rice, four *OsCAF1* genes (*OsCAF1A*, *OsCAF1B*, *OsCAF1G*, and *OsCAF1H*) have been identified, with OsCAF1B predominantly localized to processing bodies (PBs) under normal conditions, whereas the other OsCAF1 proteins remain in the cytoplasm [15,23]. Analysis of the expression patterns has revealed that these *OsCAF1* genes are involved in various abiotic stress responses [23]. However, while OsCAF1B plays a critical role in developmental processes and cold stress tolerance, the functions and subcellular localization of other OsCAF1 proteins in rice remain largely unexplored. This study investigates the role of OsCAF1A in rice heat stress tolerance and the mechanisms regulating its subcellular localization. By analyzing its expression patterns, functional significance, and interactions with the DEAD-box RNA helicases OseIF4AIIb and OsRH8, we aim to elucidate how post-transcriptional regulation modulates stress responses. Our findings reveal a heat stress-responsive regulatory mechanism controlling OsCAF1 localization to processing bodies, providing new insights into the dynamics of mRNA metabolism under stress conditions and identifying potential molecular targets for improving heat resilience in crops.

## 2. Results

### 2.1. High Temperature Induces Re-Localization of OsCAF1 Proteins in Rice Cells

**Figure 1:**
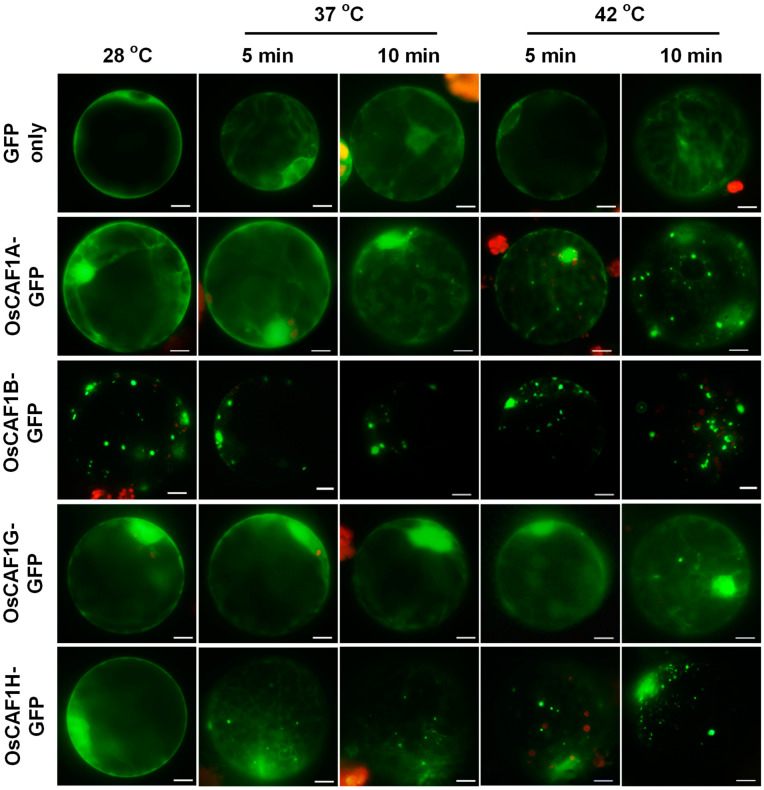
High-temperature-dependent subcellular localization of OsCAF1-GFP proteins. Rice protoplasts were transfected with constructs expressing either *GFP* alone (GFP only) or *GFP* fused to *OsCAF1A*, *OsCAF1B*, *OsCAF1G*, or *OsCAF1H*. After incubation at normal conditions (28 °C) or elevated temperature (37 °C, and 42 °C) for 5 or 10 min, GFP fluorescence was observed using fluorescence microscopy. Scale bars = 10 µm.

The localization of CAF1 proteins in yeast, mammals, and humans has been well documented, providing cytological evidence supporting their roles in RNA metabolism in vivo [21,35,36]. Our previous studies revealed that OsCAF1B is the only OsCAF1 that localizes to PBs in rice cells, whereas OsCAF1A, OsCAF1G, and OsCAF1H are dispersed throughout the cytoplasm and nucleus under normal conditions [15,23]. To further investigate the subcellular localization of OsCAF1A under high-temperature conditions, we generated N-terminal translational fusions of *OsCAF1A* with a green fluorescent protein (*GFP*), driven by the *35S* promoter of the *cauliflower mosaic virus* (*2X35S: OsCAF1A-GFP*). This construct was expressed in rice protoplasts at 28 °C and then subjected to either 37 °C or 42 °C for 5 and 10 min, after which the fluorescent signal distribution was analyzed. Under control conditions (28 °C), OsCAF1A-GFP exhibited diffuse cytoplasmic localization, consistent with previous reports [23]. However, exposure to elevated temperatures induced distinct cytoplasmic foci associated with OsCAF1A-GFP at 37 °C and 42 °C after 5 min (Figure 1). Prolonged exposure to these temperatures for 10 min led to more foci forming within the protoplasts (Figure 1). In the control experiments, protoplasts expressing *GFP* alone (GFP only) did not display any cytoplasmic foci (Figure 1). Furthermore, OsCAF1B-GFP was consistently localized to distinct cytoplasmic foci, irrespective of heat treatment (Figure 1). The localizations of OsCAF1G-GFP and OsCAF1H-GFP were also dispersed throughout the cytoplasm of protoplast cells under normal conditions (28 °C), whereas exposure to 37 °C and 42 °C led to an increase in distinct cytoplasmic foci (Figure 1). Notably, the formation of cytoplasmic foci occurred more rapidly in OsCAF1H-GFP than in OsCAF1A-GFP or OsCAF1G-GFP under heat stress (Figure 1).

To corroborate the findings of the protoplast transient expression system, we further investigated the temperature-mediated differential subcellular localization of OsCAF1s in protoplasts derived from *2X35S: OsCAF1s-GFP* and *2X35S: GFP* transgenic rice plants. In the GFP only (as a control), the fluorescence was observed in the cytoplasm without foci formation, regardless of the temperature (28 °C or 42 °C). OsCAF1B-GFP consistently localized to distinct cytoplasmic foci under normal and heat stress conditions in transgenic rice plants. In contrast, OsCAF1A-GFP and OsCAF1H-GFP exhibited cytoplasmic dispersion at 28 °C, but formed distinct cytoplasmic foci at 42 °C following heat exposure (Supplementary Figure S1). Consistent with the transient assay results, OsCAF1H-GFP re-localized to the cytoplasmic foci more rapidly than did OsCAF1A-GFP and OsCAF1G-GFP, showing a significant relocation within 10 min in protoplasts (Supplementary Figure S1). These results indicate that the subcellular localization of OsCAF1A-GFP, OsCAF1G-GFP, and OsCAF1H-GFP responded to temperature variations, with foci formation occurring at higher temperatures.

### 2.2. OsCAF1 Proteins Localize to Processing Bodies and Stress Granules Under High-Temperature Conditions

**Figure 2:**
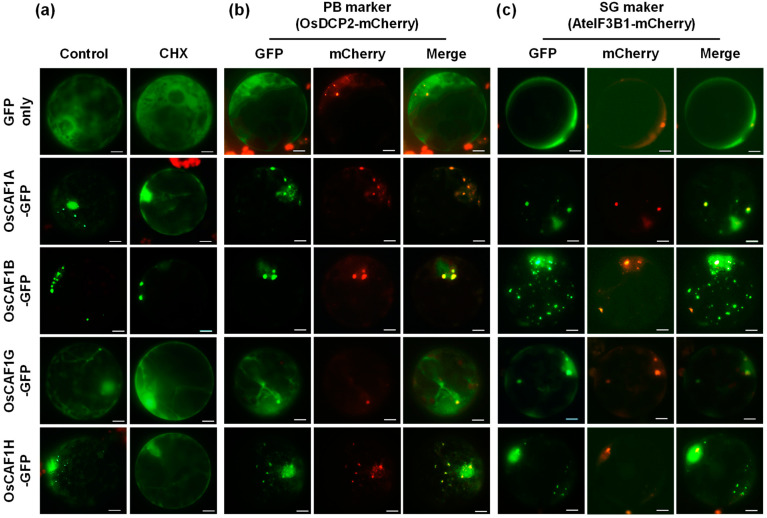
High temperature induces the re-localization of OsCAF1s-GFP to processing bodies and stress granules. (**a**) Cycloheximide (CHX) treatment reduces the formation of OsCAF1s-containing foci. Rice protoplasts expressing *OsCAF1s-GFP* (*OsCAF1A-GFP*, *OsCAF1B-GFP*, *OsCAF1G-GFP*, or *OsCAF1H-GFP*) were treated with 35 μM CHX or dimethyl sulfoxide (DMSO, control) at 28 °C for 15 min, followed by heat treatment at 42 °C for 30 min. GFP fluorescence distribution was analyzed using fluorescence microscopy. (**b**,**c**) Under high-temperature conditions, OsCAF1s-GFP fully and partially colocalizes with the processing body (PB) and the stress granule (SG) markers, respectively. Rice protoplasts were co-transfected with *OsCAF1s-GFP* and either *OsDCP2-mCherry* (the PB marker) or *AteIF3B1-mCherry* (the SG marker). After incubation at 42 °C for 10 min, GFP and mCherry fluorescence were analyzed using fluorescence microscopy. *GFP* alone served as a control. Sale bars = 10 μm.

Previously published expression data [37] have indicated that the exogenous application of chemicals can modulate the formation and dispersal of PBs and SGs in plant cells. Cycloheximide (CHX), which inhibits the elongation phase of protein synthesis, sequesters mRNA within polysomes and decreases the formation of cytosolic ribonucleoprotein granules, including PBs and SGs, in plant cells [37]. To investigate whether the OsCAF1A-containing cytoplasmic foci that appear in response to high temperatures are related to these ribonucleoprotein granules, rice protoplasts expressing OsCAF1A-GFP proteins were treated with CHX and exposed to 42 °C for 30 min. Analysis of the effect of CHX treatment on OsCAF1A-containing foci revealed a significant reduction compared to the untreated controls under high-temperature conditions (Figure 2a). Additionally, CHX treatment blocked the assembly of heat stress-induced foci in other OsCAF1 proteins, confirming that these foci were related to PBs or SGs (Figure 2a).

To confirm whether foci containing OsCAF1A-GFP induced by high temperatures represented PBs, we co-expressed OsCAF1A-GFP with the PB marker OsDCP2-mCherry [38] in rice protoplasts. Under high-temperature conditions (42 °C for 30 min), OsCAF1A-GFP was found to be fully colocalized with OsDCP2-mCherry (Figure 2b). Similar colocalization patterns were observed for the OsCAF1G-GFP and OsCAF1H-GFP proteins (Figure 2b). Furthermore, OsCAF1B-GFP colocalized with the PB marker under both normal and high-temperature conditions. (Figure 2b). Next, we investigated whether OsCAF1A-GFP was targeted to SGs under heat stress. We co-expressed the SG marker AteIF3B1-mCherry [39], fused with mCherry and OsCAF1A-GFP in the protoplasts. Under high-temperature conditions, OsCAF1A-GFP exhibited partial colocalization with AteIF3B1-mCherry, indicating an incomplete association with SGs (Figure 2c). A similar pattern of partial colocalization was observed for other OsCAF1-GFP proteins with the SG marker following heat stress exposure (Figure 2c). Protoplasts expressing GFP only (the control group) did not exhibit foci colocalization with the PB or SG markers (Figure 2b,c). These findings suggest that OsCAF1 is predominantly associated with PBs under high-temperature conditions.

### 2.3. OseIF4AIIb and OsRH8 Interacts with OsCAF1 Proteins

To identify potential proteins that may interact with OsCAF1s and regulate their subcellular localization, total proteins were extracted from embryo calli expressing *2X35S: OsCAF1A-GFP* and *2X35S: GFP* (control) transgenic lines (Supplementary Figure S2). These proteins were subjected to co-immunoprecipitation (co-IP) using the GFP-trapping method [40], followed by LC-MS/MS analysis [41]. Proteins interacting with OsCAF1A were specifically identified in OsCAF1A-GFP overexpressing transgenic lines, using stringent criteria and a false discovery rate of <1% for peptide identification. In addition to the core components of the rice CCR4-NOT complex (Table 1), several putative rice proteins with top peptide spectrum matches (PSMs) exceeding 20 were identified as highly abundant OsCAF1A-interacting partners (Table 2).

As previously reported [42,43,44], two DEAD-box helicases, eIF4A2 and DDX6, have been identified as interacting with CAF1 and exert opposing effects on human CAF1 protein activity [33]. Using IP-MS analysis, OseIF4AIIb and the DDX6 ortholog in rice, OsRH8 were identified as highly abundant interacting partners of OsCAF1A (Table 2). Both proteins displayed high sequence coverage and numerous unique peptides in the dataset, indicating their status as high-confidence candidate interacting proteins.

Various combinations of nEYFP- and cEYFP-fused proteins were co-expressed in onion epidermal cells via particle bombardment to validate these interactions. The interaction patterns between OseIF4AIIb, OsRH8, and OsCAF1A in onion epidermal cells were consistent with previous observations (Supplementary Figure S3).

**Figure 3:**
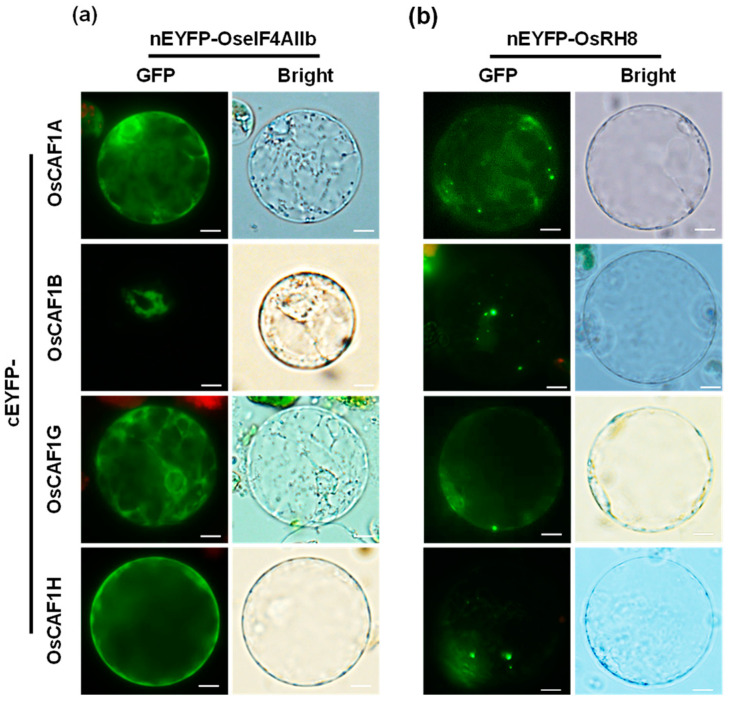
Interaction between OsCAF1s and OseIF4AIIb or OsRH8 proteins. BiFC assays were conducted to assess the interactions between OsCAF1s and OseIF4AIIb (**a**) or OsRH8 (**b**) in rice protoplasts. The N-terminal fragment of *EYFP* (*nEYFP*) was fused to *OseIF4AIIb* or *OsRH8*, whereas the C-terminal fragment of *EYFP* (*cEYFP*) was fused to *OsCAF1s*. Rice protoplasts were co-transfected with *nEYFP-OseIF4AIIb*, *nEYFP-OsRH8*, and *cEYFP-OsCAF1s.* Fluorescence microscopy images were acquired to visualize the EYFP signals, with green fluorescence indicating positive interactions. Sale bars = 10 µm.

To confirm these interactions between OsCAF1s and OseIF4AIIb or OsRH8, a bimolecular fluorescence complementation (BiFC) assay was performed. The N-terminal fragment of EYFP (nEYFP) was fused to OseIF4AIIb or OsRH8, and the C-terminus of EYFP (cEYFP) was fused to OsCAF1s. Various combinations of nEYFP- and cEYFP-fused proteins were transiently co-expressed in rice protoplasts. EYFP fluorescence was detected only in the cytoplasm upon the co-expression of nEYFP-OseIF4AIIb and cEYFP-OsCAF1s (Figure 3a). Interaction signals between OsRH8 and OsCAF1s were detected in the cytoplasm and at prominent cytoplasmic foci (Figure 3b). These results indicate that both OseIF4AIIb and OsRH8 interact with OsCAF1.

### 2.4. Subcellular Localization of OseIF4AIIb and OsRH8

**Figure 4:**
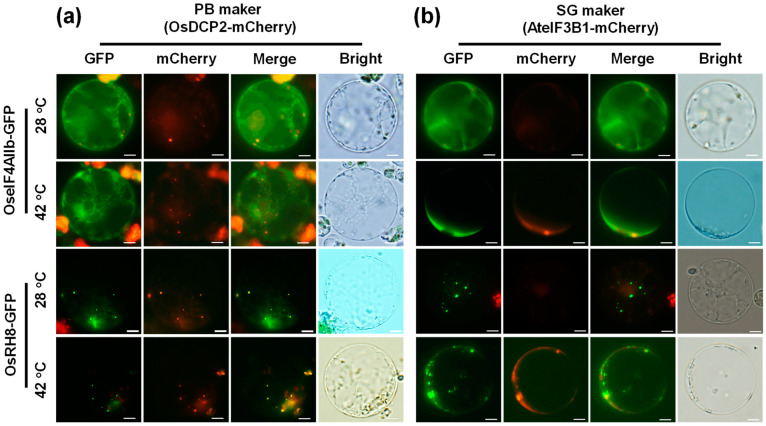
Subcellular localization of OseIF4AIIb-GFP and OsRH8-GFP proteins. Rice protoplasts were transfected with constructs expressing *OseIF4AIIb-GFP* or *OsRH8-GFP* in combination with either the processing body (PB) marker *OsDCP2-mCherry* (**a**) or the stress granule (SG) marker *AteIF3B1-mCherry* (**b**). The protoplasts were incubated under normal (28 °C) or heat stress (42 °C) conditions for 30 min, and the subcellular localization of OseIF4AIIb-GFP and OsRH8-GFP was analyzed using fluorescence microscopy. Sale bars = 10 μm.

eIF4A2, a cytoplasmic paralog of eIF4A, has been reported to function redundantly in translation initiation by associating with eIF4G within the eIF4F complex [45]. In contrast, DDX6 plays a crucial role in translational regulation and is a central component of cytoplasmic mRNA degradation bodies (PBs) [14]. It has been suggested that OseIF4AIIb and OsRH8 are located differently within cells. To investigate the subcellular localization of OseIF4AIIb and OsRH8, the full-length coding sequences of either *OseIF4AIIb* or *OsRH8* were fused in-frame to the N-terminus of GFP. These constructs were expressed under the control of the *35S* promoter from the *cauliflower mosaic virus* (*2X35S*: *OseIF4AIIb-GFP* or *2X35S*: *OsRH8-GFP*). The resulting constructs were transiently co-expressed with the PB marker (OsDCP2-mCherry) or SG marker (AteIF3B1-mCherry) in rice protoplasts. Fluorescence data revealed that the OseIF4AIIb-GFP signal was consistently localized in the cytoplasm under normal and high-temperature conditions without forming cytosolic foci (Figure 4). In contrast, OsRH8-GFP signals were detected in the cytoplasm, with some foci fully colocalized with OsDCP2-mCherry under normal conditions (Figure 4). Additionally, after 30 min of high-temperature exposure, most OsRH8-GFP signals completely overlapped with OsDCP2-mCherry and were partially associated with AteIF3B1-mCherry (Figure 4). These results indicate that OseIF4AIIb was consistently localized in the cytoplasm regardless of heat treatment, whereas OsRH8 was found in both the cytoplasm and PBs, with elevated temperatures promoting its predominant localization to PBs.

To further investigate whether the expression of OsCAF1-interacting genes is a response to heat stresses, RT-qPCR was used to determine the mRNA levels of *OseIF4AIIb* and *OsRH8* genes in the roots and shoots of 2-week-old rice seedlings subjected to heat (42 °C) treatments. This result indicates a slight induction of *OseIF4AIIb* mRNA expression in the roots following heat exposure (Supplementary Figure S4a). In contrast, *OsRH8* mRNA levels were elevated 6.1-fold in the roots after 3 h and 2.2-fold in the shoots after 6 h of exposure to 42 °C (Supplementary Figure S4b). These results suggest that OseIF4AIIb and OsRH8 exhibit distinct localization and differential expression patterns in response to heat stress.

### 2.5. High Temperature-Mediated Localization of P-Bodies of the OsCAF1s Is Reduced by OseIF4AIIb and Promoted by OsRH8

**Figure 5:**
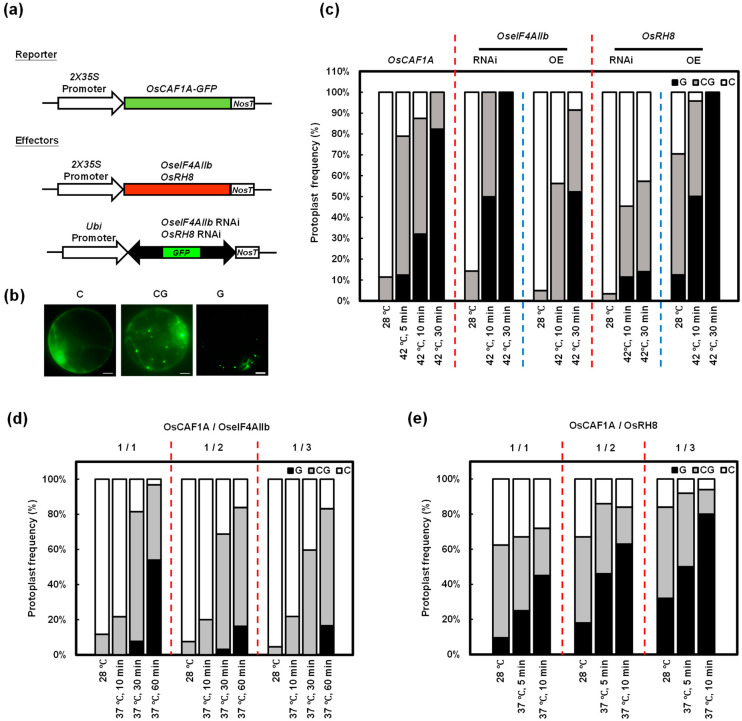
OsIF4AIIb suppresses the re-localization of OsCAF1A to cytoplasmic foci, while OsRH8 promotes this process. (**a**) Schematic representation of the reporter and effector constructs used in the experiments. (**b**) Fluorescence microscopy images showing the subcellular localization patterns of GFP-fused OsCAF1A proteins. “C” indicates GFP signals dispersed in the cytoplasm, “G” represents GFP signals localized in foci, and “CG” denotes GFP signals present in both the cytoplasm and foci. (**c**) Quantification of the distribution patterns of OsCAF1A-GFP in rice protoplasts co-transfected with OsCAF1A effector and respective reporter plasmids. Protoplasts were incubated under normal (28 °C) and heat stress (42 °C) conditions for the indicated time points. The proportions of protoplasts exhibiting each GFP distribution pattern were quantified (n > 100). (**d**,**e**) Quantification of the distribution patterns of OsCAF1A-GFP in rice protoplasts co-transfected with 10 µg of *2X35S:OsCAF1A-GFP* and varying amounts of either *2X35S:OseIF4AIIb* or *2X35S:OsRH8*, respectively. Protoplasts were subjected to normal (28 °C) and heat stress (37 °C or 42 °C) conditions for the indicated time points. The proportions of protoplasts displaying different GFP distribution patterns were quantified (n > 100).

In mammalian cells, CAF1 plays a vital role in PB formation [35]. Meijer et al. [33] demonstrated that two DEAD-box helicases, eIF4A2 and DDX6, exert opposing effects on human CAF1 protein function. Based on these findings, we investigated the roles of OseIF4AIIb and OsRH8 (the DDX6 orthologs in rice) in regulating the subcellular localization of OsCAF1 proteins in response to high temperatures. To address this question, rice protoplasts were co-transfected with various constructs: OsCAF1A-GFP, overexpression (OE) *OseIF4AIIb*, knockdown (RNAi) *OseIF4AIIb*, overexpression (OE) *OsRH8*, and knockdown (RNAi) *OsRH8* (Figure 5a). The distribution of OsCAF1A-GFP was monitored during incubations at 28 °C and subsequent exposure to 42 °C at the indicated time points. OsCAF1A-GFP signals were categorized as follows: dispersed in the cytoplasm (“C”), associated with foci (“G”), and present in both the cytoplasm and foci (“CG”) (Figure 5b).

At 28 °C, OsCAF1A-GFP was distributed throughout the cytoplasm of most protoplasts, with only 12% showing OsCAF1A-GFP in both the cytoplasm and PBs (Figure 5c). In contrast, at 42 °C, protoplasts displayed significant accumulation of OsCAF1A-GFP in PBs. Specifically, 87% of protoplasts exposed to 42 °C for 10 min and 100% of those exposed to 42 °C for 30 min exhibited OsCAF1A-GFP foci, with 32% and 82% of protoplasts, respectively, showing OsCAF1A-GFP foci exclusively (Figure 5c, left). In protoplasts co-transfected with OsCAF1A-GFP and *OseIF4AIIb* RNAi constructs, OsCAF1A-GFP showed enhanced accumulation in PBs under 42 °C conditions. All protoplasts (100%) exposed to 42 °C for 10 and 30 min contained OsCAF1A-GFP foci, with 49% and 100% of protoplasts, respectively, exhibiting OsCAF1A-GFP foci exclusively (Figure 5c, middle). In contrast, when protoplasts were co-transfected with the *OseIF4AIIb* OE construct, the high-temperature-induced accumulation of OsCAF1A-GFP in the PBs was suppressed. Only 57% and 92% of protoplasts exposed to 42 °C for 10 and 30 min, respectively, displayed OsCAF1A-GFP foci. None of the protoplasts exposed to 42 °C for 10 min exhibited foci exclusively, and only 53% of protoplasts exposed to 42 °C for 30 min showed foci exclusively (Figure 5c, middle). On the other hand, co-transfection of OsCAF1A-GFP with *OsRH8* overexpression (OE) constructs in rice protoplasts resulted in an increased percentage of protoplasts containing OsCAF1A-GFP foci under both 28 °C and 42 °C conditions compared to protoplasts expressing only OsCAF1A-GFP (Figure 5c, right). Conversely, the knockdown of *OsRH8* significantly reduced the number of protoplasts containing OsCAF1A-GFP foci, even under high-temperature conditions (Figure 5c, right). To confirm the effects of OseIF4AIIb and OsRH8 on the subcellular localization of OsCAF1A, rice protoplasts were co-transfected with OsCAF1A-GFP and either *OseIF4AIIb* OE or *OsRH8* OE constructs at various molar ratios. Increasing amounts of OseIF4AIIb decreased the formation of high-temperature-induced OsCAF1A-GFP foci, whereas higher levels of OsRH8 increased the formation of OsCAF1A-GFP foci under high-temperature conditions (Figure 5d,e).

Similar results were observed in rice protoplasts co-transfected with OsCAF1H-GFP and various combinations of constructs, including *OseIF4AIIb* OE, *OseIF4AIIb* RNAi, *OsRH8* OE, and *OsRH8* RNAi, or with different molar ratios of *OseIF4AIIb* OE and *OsRH8* OE (Supplementary Figure S5). These results suggest that OseIF4AIIb inhibits the association of OsCAF1 with PBs, whereas OsRH8 enhances their accumulation, highlighting their opposing roles in regulating OsCAF1 localization under high-temperature stress in rice.

### 2.6. OsCAF1A mRNA Is Highly Expressed in Rice Seedlings and Further Induced Under High-Temperature Conditions

**Figure 6:**
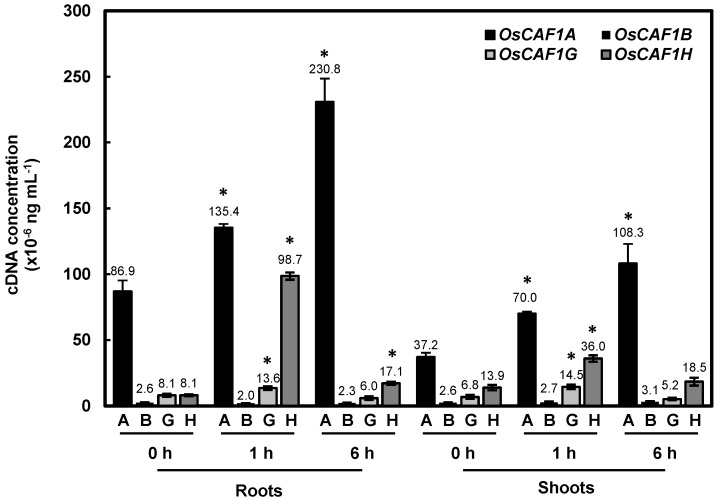
*OsCAF1A* mRNA is abundantly expressed in rice seedlings. Ten-day-old seedlings were treated at 42 °C for 0, 1, and 6 h, followed by absolute quantitative RT-PCR analysis using *OsCAF1* gene-specific primers. *OsCAF1* plasmid DNAs were used as a control to compare the mRNA levels of various *OsCAF1* genes. Error bars indicate the SE of three replicates. Significant differences from 0 h were assessed using Student’s *t*-test (* *p* < 0.05).

In our previous study, four expressed *OsCAF1* genes were identified in rice: *OsCAF1A*, *OsCAF1B*, *OsCAF1G*, and *OsCAF1H*. High-temperature conditions induce the expression of *OsCAF1A*, whereas *OsCAF1H* exhibits a transient response to heat stress [23]. To compare the transcription levels of these *OsCAF1* genes under normal conditions and their accumulations in response to high temperatures, rice seedlings were exposed to 42 °C for varying durations, followed by an absolute quantitative RT-PCR analysis. The threshold cycle (Ct) values obtained from qPCR against serial dilutions of individual *OsCAF1* cDNA-containing plasmids were used to generate a standard curve for each sample (Supplementary Figure S6). These standard curves allowed precise quantification of *OsCAF1A* mRNA levels by interpolating Ct values obtained from cDNA samples, ensuring accurate comparisons across different conditions. Consistent with the previous reports [23], *OsCAF1H* mRNA levels showed transient induction at 1 h, followed by a decrease to basal levels after 6 h of high-temperature exposure. Under normal conditions, *OsCAF1A* mRNA levels were significantly higher than the mRNA levels of *OsCAF1B*, *OsCAF1G*, and *OsCAF1H* in both the roots and shoots of rice seedlings (Figure 6). Moreover, *OsCAF1A* mRNA levels in rice seedlings exhibited a marked increase within 1 h of high-temperature exposure, with continued accumulation observed throughout the 6 h period (Figure 6). The significantly higher abundance of *OsCAF1A* mRNA under both normal and high-temperature conditions, compared to other *OsCAF1* genes, suggests a critical role for OsCAF1A in the heat stress response of rice seedlings.

### 2.7. OsCAF1A Is Required for Rice Seedling Response to High Temperature

**Figure 7:**
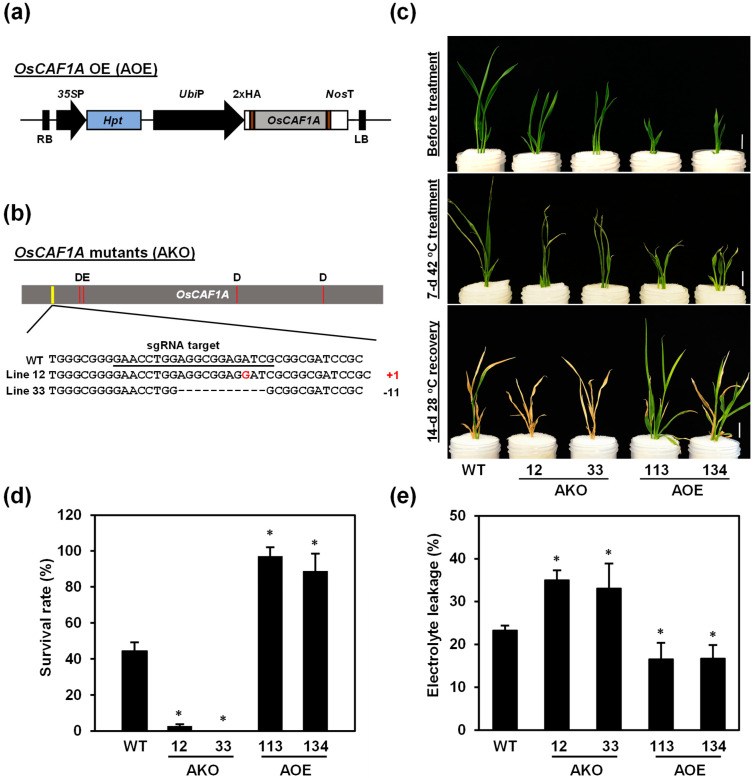
*OsCAF1A* expression is required for rice seedlings to exhibit heat tolerance. Schematic representation of *OsCAF1A* mutation sites (**a**) and *OsCAF1A* overexpression cassette (**b**). Two *OsCAF1A* mutant lines (lines 12 and 33) were generated using the CRISPR/Cas9 system. The *OsCAF1A* DNA sequences of the mutated sites were aligned with the genomic *OsCAF1A* DNA sequences of the wild-type (WT) (underline: sgRNA target; “+”: inserted base; “−”: deleted base). *OsCAF1A* cDNA was inserted downstream of the *Ubiquitin 1* (*Ubi*) promoter to generate transgenic rice. (**c**) Examination of heat tolerance of rice seedlings. Seven-day-old seedlings of WT, two *OsCAF1A*-mutant lines (AKO-12 and AKO-33), and two independent *OsCAF1A*-overexpression lines (AOE-113 and AOE-134) were incubated at 42 °C for 7 days and then allowed to recover at 28 °C for 14 days. Plant morphology was photographed. Sale bars = 1 cm. (**d**) Survival rate of the seedlings in the experiment (**b**) was determined. Error bars indicate the SE of 30 individual seedlings for each line. (**e**) Electrolyte leakage was determined from 2-week-old seedlings incubated at 42 °C for 5 days. Error bars indicate the SE of 10 individual seedlings for each line. Significant differences from the WT were determined using Student’s *t*-test (* *p* < 0.05).

To determine the potential function of *OsCAF1A* in the heat stress response of rice, two homozygous loss-of-function rice mutants of *OsCAF1A*, AKO-12, and AKO-33 (Figure 7b), were generated using the CRISPR/Cas9 approach. The *OsCAF1A* coding region in AKO-12 and AKO-33 exhibited an insertion of one nucleotide and deletion of 11 nucleotides, respectively, resulting in a frameshift and a premature termination codon (Supplementary Figure S7a). The *OsCAF1A* overexpression transgenic rice lines were generated using *OsCAF1A* cDNA fused downstream of the maize ubiquitin gene (*Ubi*) promoter and two independent homozygous lines with high levels of *OsCAF1A* mRNA, named AOE-113 and AOE-134 (Figure 7a, Supplementary Figure S7b), were obtained. *OsCAF1A*-mutant and -overexpressing transgenic lines were propagated to the T3 to T5 generations and displayed morphological defects at the vegetative stages. Shoot length in the *OsCAF1A* mutant lines was slightly greater than that in WT plants at the two-week-old seedlings stage, whereas the *OsCAF1A*-overexpression lines exhibited significantly reduced shoot length compared to the WT (Supplementary Figure S7c,d).

To determine whether *OsCAF1A* is involved in the high-temperature response mechanism of rice, one-week-old hydroponically grown seedlings were incubated at 42 °C for 7 days and subsequently kept under normal conditions for 2 weeks to assess recovery. During the high-temperature period, the two independent *OsCAF1A*-mutated lines failed to grow properly, showing leaf desiccation and wilting, ultimately resulting in their death (Figure 7c). In contrast, *OsCAF1A*-overexpression seedlings displayed sustained growth and retained a vibrant green phenotype (Figure 7c). Survival rates were assessed after a 14 d recovery period at 28 °C, revealing that the two *OsCAF1A*-mutated lines had lower survival rates, 2.8% and 0%, respectively, compared with 44.4% in the WT (Figure 7d). Conversely, the survival rates of the AOE-113 and AOE-134 lines were significantly higher than that of the WT, ranging from 97.2% to 88.9% (Figure 7d). To further assess heat tolerance, we measured electrolyte leakage, a common indicator of membrane damage in plants [30], in two-week-old seedlings subjected to 42 °C for 5 days. The results indicated lower electrolyte leakage in the *OsCAF1A*-overexpression lines, but higher in *OsCAF1A*-mutant lines compared to WT plants (Figure 7e). These findings underscore the critical role of *OsCAF1A* in conferring heat stress tolerance to rice seedlings.

### 2.8. OsCAF1A Promotes Rice Seedling Growth Under Sublethal High-Temperature Treatment

**Figure 8:**
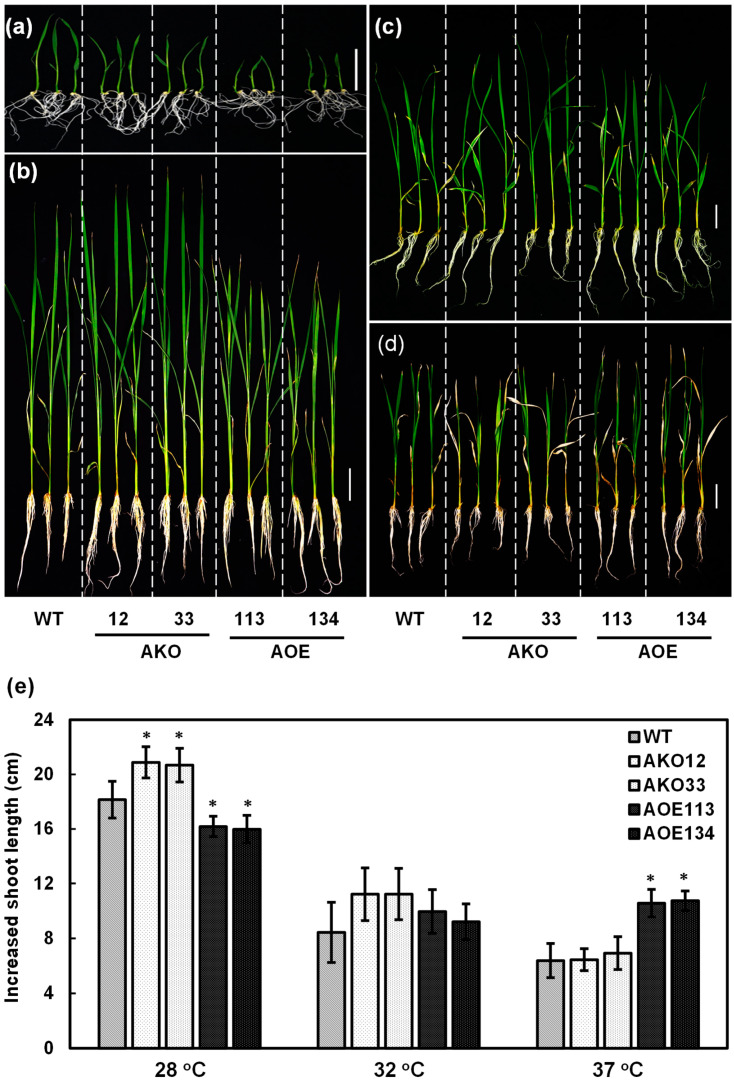
Enhanced sublethal heat stress tolerance in rice seedlings with increased *OsCAF1A* expression. (**a**) Seeds from wild-type (WT), two *OsCAF1A* mutant lines (AKO-12 and AKO-33), and two independent *OsCAF1A*-overexpression lines (AOE-113 and AOE-134) were germinated at 28 °C for 7 days. (**b**–**d**) Seven-day-old seedlings of each line were further exposed to 28 °C (**b**), 32 °C (**c**), and 37 °C (**d**) for an additional 14 days. The phenotypes of the seedlings are shown. Sale bars = 3 cm. (**e**) Increased shoot length of seedlings was measured for each line across the three different temperature conditions. Error bars represent the SE of 30 individual seedlings. Significant differences from WT were assessed using Student’s *t*-test (* *p* < 0.05).

The loss-of-function mutation in *OsCAF1A* impaired heat stress tolerance in rice seedlings (Figure 7). To further elucidate the role of *OsCAF1A* in the adaptation of rice seedlings to elevated temperatures, we assessed seedling growth under sublethal heat stress. One-week-old seedlings from WT, *OsCAF1A*-mutant (AKO) lines, and *OsCAF1A*-overexpressing (AOE) lines were exposed to either control conditions (28 °C) or sublethal heat stress conditions (32 °C and 37 °C) for an additional 14 days. Increased shoot lengths were recorded in all treated seedlings. At the 7-day-old seedling stage, the WT plants exhibited the greatest shoot length, followed by the mutant lines, with the overexpression lines displaying the shortest shoot length (Figure 8a). However, after an additional 14 days under normal conditions (28 °C), the mutant lines displayed increased shoot length, while the overexpression lines showed less growth than WT plants (Figure 8e). Consequently, the mutant lines surpassed the WT in seedling height, while plants in the overexpression lines remained the shortest (Figure 8b). Exposure to 32 °C for an additional two weeks significantly reduced shoot length growth across all lines. The increase in shoot length in both the mutant lines and the overexpression lines was comparable to that of the WT plants (Figure 8c,e). However, under 37 °C treatment, the overexpression lines exhibited a greater increase in shoot length compared to the WT and mutant lines, resulting in a slightly greater shoot length in the overexpression lines (Figure 8d,e). These results indicated that *OsCAF1A* expression enhanced the adaptability of rice seedlings to sublethal temperatures.

## 3. Discussion

Understanding rice adaptation to abiotic stress is essential for developing climate-resilient crops considering that high-temperature stress currently poses a major threat to rice growth and development in tropical regions [3,4]. mRNA degradation is essential for gene expression regulation by controlling mRNA levels, eliminating defective or superfluous transcripts, maintaining cellular homeostasis, and facilitating rapid responses to environmental changes [14,16,46,47]. Processing bodies (PBs), which concentrate key components of the mRNA degradation machinery, are implicated in both mRNA decay and translational repression [26,27]. Members of the DEAD-box family of RNA helicases, which are involved in transcription, translation, and mRNA decay, play a crucial role in regulating gene expression at multiple levels [31]. This study demonstrated that two DEAD-box RNA helicases, OseIF4AIIb and OsRH8, are critical for modulating the recruitment of OsCAF1 proteins to PBs in heat-stressed rice. Furthermore, the results of our research show that OsCAF1A is crucial for conferring heat stress tolerance in rice seedlings.

### 3.1. OsCAF1 Proteins as Components of PBs in Rice Under High-Temperature Conditions

The subcellular localization of proteins is a critical determinant of their function, which affects cellular processes and the overall response of an organism to environmental stimuli. Proteins are often strategically localized in specific cellular compartments where they perform specialized functions, ensuring the efficient execution of cellular functions [48]. PBs play crucial roles in mRNA decay and translational repression [26]. In plants, the typical components of PBs include many RNA-binding proteins (RBPs) and key factors for mRNA decay, including de-capping subunits (e.g., DCP1/2, exoribonuclease (e.g., XRN1) and de-adenylation factors (e.g., CCR4) [49].

The CCR4-NOT complex is well-known for its role in the mRNA poly(A) shortening mechanism mediated by the de-adenylase activity of CAF1. CAF1 is essential for initiating mRNA decay and forming PBs. In yeast, the deletion of *Pop2*, a *CAF1* ortholog, reduces the accumulation of PB components such as DCP1 and DCP2 under stress conditions [34]. Similar observations in human cells demonstrate that *CAF1* knockdown diminishes the formation of PB foci, underscoring its role in PB assembly [29].

In rice, the four OsCAF1 proteins exhibited distinct localization patterns: OsCAF1B-GFP localized almost exclusively to the PBs, whereas the other OsCAF1-GFP proteins were primarily located in the cytoplasm under normal conditions (Figure 1 and Figure S1). Our results demonstrated that OsCAF1A, OsCAF1G, and OsCAF1H were predominantly localized to PB markers, such as OsDCP2, following heat stress (Figure 2b). These findings are consistent with observations of POP2, a yeast CAF1 ortholog that also relocates to PBs under stress conditions [34]. Since CHX prevents the accumulation of free mRNA in cytoplasmic foci [37], the assembly of OsCAF1-containing foci suggests that OsCAF1 proteins are specifically recruited to PBs under heat stress, likely in response to the accumulation of untranslated mRNAs (Figure 2a). OsCAF1B consistently accumulated in PBs under normal and high-temperature conditions, suggesting its role in maintaining basal mRNA degradation processes (Figure 2b). OsCAF1A exhibits the most pronounced upregulation under heat stress, accompanied by its rapid and dynamic re-localization to PBs. This observation suggests that OsCAF1A may play a central role in stress adaptation by promoting the degradation of untranslated mRNAs within PBs, thereby preventing the accumulation of nonfunctional transcripts. Such targeted mRNA decay ensures the efficient turnover of stress-responsive transcripts, enabling precise regulation of gene expression to optimize cellular homeostasis and enhance heat stress resilience.

### 3.2. Mechanistic Insights into the Regulation of OsCAF1 Protein Re-Localization by OseIF4AIIb and OsRH8

Notably, while the subcellular localization of CAF1 proteins can be altered under specific conditions, the recruitment of CAF1 to PBs is also influenced by interacting proteins, including DEAD-box helicase proteins [34]. In humans, the DEAD-box helicases DDX6 and eIF4A2 modulate CAF1 activity in opposite ways, thereby influencing mRNA stability and decay [32,33]. Our study identified two DEAD-box RNA helicases in rice, OseIF4AIIb, and OsRH8, as key interactors with OsCAF1 proteins, exhibiting distinct localization patterns in rice cells and opposing effects on OsCAF1 re-localization under heat stress conditions.

In human cells, eIF4A2 inhibits CNOT7-mediated de-adenylation, resulting in mRNAs with longer poly(A) tails that prevent their rapid degradation [32]. This inhibition is crucial for regulating mRNA stability and translation as it prevents the rapid degradation of specific mRNAs, thereby modulating gene expression at the post-transcriptional level [33]. Our study extends this understanding by showing that the rice ortholog OseIF4AIIb remains cytoplasmic under all conditions (Figure 4) and interacts with OsCAF1 proteins exclusively in the cytoplasm (Figure 3). OseIF4AIIb inhibited the re-localization of OsCAF1 proteins to PBs under heat stress (Figure 5, Supplementary Figure S5), which likely stabilizes mRNAs by preventing their degradation in PBs. Thus, OseIF4AIIb acts as a translational repressor, regulating mRNA stability and translation through multiple mechanisms.

OsRH8, a DEAD-box helicase orthologous to DDX6, is localized in the cytoplasm and cytoplasmic foci under normal conditions but relocates to PBs under heat stress (Figure 4). Furthermore, OsRH8 interacted with OsCAF1 proteins (Figure 3) and facilitated their re-localization to PBs in response to elevated temperatures (Figure 5, Supplementary Figure S5). This is consistent with previous findings that Dhh1p, a DEAD-box helicase orthologous to DDX6 in yeast, efficiently recruits Pop2p to proteins, particularly during glucose deprivation in yeast [34]. The differential effects of OseIF4AIIb and OsRH8 on OsCAF1 localization highlight the intricate regulation of mRNA metabolism. OseIF4AIIb inhibition of OsCAF1 re-localization stabilizes mRNAs, whereas OsRH8 promotion of OsCAF1 recruitment to PBs facilitates mRNA decay, suggesting that a balance between mRNA stabilization and degradation is essential for optimizing gene expression. This dynamic regulation is critical for effective stress responses and significantly affects plant growth and development.

### 3.3. OsCAF1A Confers Heat Stress Tolerance but Compromises Growth in Rice

Gain- and loss-of-function analyses have revealed the importance of PB components in plant development and stress responses [14,15,16]. Rice contains four distinct OsCAF1 proteins, OsCAF1A, OsCAF1B, OsCAF1G, and OsCAF1H. Among these, OsCAF1B has been specifically recognized as a component of PBs and is involved in rice development and cold stress tolerance. OsCAF1H demonstrated a distinct, transient response to elevated temperatures and rapid re-localization to PBs (Figure 1 and Figure 2), accompanied by a transient increase in mRNA levels shortly after heat exposure, with the levels returning to baseline after continued high-temperature treatment (Figure 6). Notably, the mRNA level of *OsCAF1A* was more abundantly expressed than that of the other *OsCAF1* genes in rice seedlings under normal conditions and exhibited sustained upregulation throughout heat stress (Figure 6). These observations suggest that OsCAF1A plays a more prominent and sustained role in mediating heat stress tolerance in rice than OsCAF1H.

Ectopic expression of *OsCAF1A* significantly increased the survival rate of rice seedlings under heat stress (Figure 7) and enhanced seedling growth under sublethal heat stress conditions (Figure 8), suggesting that enhancing *OsCAF1A* expression is a promising strategy for improving heat stress tolerance in rice. However, constitutive overexpression of *OsCAF1A* caused severe stunting of rice seedlings under normal conditions (Figure 7c and Figure 8; Supplementary Figure S7c,d). In contrast, *OsCAF1A* mutants exhibited no significant differences in growth and development under the same conditions (Figure 7c and Figure 8; Supplementary Figure S7c,d), suggesting a potential redundancy among OsCAF1 family members in maintaining mRNA homeostasis. These findings indicate that while OsCAF1A contributes to stress tolerance, excessive de-adenylation activity may disrupt transcriptome balance, negatively impacting growth.

OsCAF1A functions as a de-adenylase, catalyzing the removal of poly(A) tails from mRNAs and facilitating their degradation [23]. While mRNA turnover is essential for gene expression regulation, excessive degradation due to high *OsCAF1A* expression could reduce the stability of transcripts critical for growth. One possible explanation for this phenotype is that OsCAF1A preferentially targets specific mRNAs involved in growth regulation. Identifying these target transcripts is crucial for understanding the molecular basis of OsCAF1A-mediated growth defects. A potential approach is to use the HyperADARcd (Hyperactive Adenosine Deaminase Acting on RNA catalytic domain) system [50], which enables RNA editing near OsCAF1A-bound transcripts. By fusing OsCAF1A to HyperADARcd, A-to-I editing events can be introduced in OsCAF1A-associated RNAs, facilitating their identification via RNA-seq. Subsequent validation through expression analysis in *OsCAF1A* overexpression, mutant, and wild-type lines, coupled with poly(A) tail assays, could determine whether OsCAF1A directly accelerates their decay.

A stress-inducible promoter may better control *OsCAF1A* expression, mitigating its downsides at high constitutive levels while retaining its stress-protective role. This approach would allow *OsCAF1A* to enhance heat stress tolerance without compromising normal growth. Consequently, such a strategy holds significant potential for improving stress resilience in rice and other crops while minimizing trade-offs in plant development [16,30].

## 4. Materials and Methods

### 4.1. Plant Materials and Growth Conditions

The rice cultivar *Oryza sativa* L. cv Tainung 67 (TNG67) and its transgenic lines, namely, two mutant *OsCAF1A* lines (AKO), two lines overexpressing the *OsCAF1A* gene (AOE) driven by the maize *Ubiquitin 1* (*Ubi*) promoter, and lines expressing *2X35S: OsCAF1-GFP* were used as genetic materials in this study. The de-hulled seeds were surface-sterilized with 3% NaOCl for 30 min, followed by thorough rinsing with sterile water. Sterilized seeds were sown on half-strength Murashige and Skoog agar medium containing 3% (*w*/*v*) sucrose and incubated at 28 °C under continuous light for 7 days. After germination, rice seedlings were subjected to heat tolerance testing. For hydroponic cultivation, seedlings were transferred to 50 mL Falcon tubes containing half-strength Kimura B nutrient solution (pH 4–5), which was refreshed daily.

### 4.2. Plasmid

Plasmid pMDC85 [23] was used to construct the C-terminal GFP fusion constructs. To construct mCherry destination vectors, the GFP sequence in the pMDC85 plasmid was replaced with mCherry. For the bimolecular fluorescence complementation (BiFC) assay, the pSAT4-DEST-nEYFP-C1 and pSAT5-DEST-cEYFP-C1 plasmids, obtained from the Arabidopsis Biological Resource Center, were used as Gateway vectors. Additionally, the pCAMBIA vectors were sourced from CAMBIA (www.cambia.org). The sequences of primers utilized for plasmid construction are provided in Supplementary Table S1.

### 4.3. Plasmid Construction

To amplify the coding sequences of *OseIF4AIIb* and *OsRH8*, PCR was performed using specific primers (Supplementary Table S1) and Phusion High-Fidelity DNA Polymerase (New England Biolabs, Ipswich, MA, USA), with cDNA derived from rice suspension-cultured cells as the templates. The amplified products were cloned into the yT&A cloning vector (Yeastern Biotech, Taipei, Taiwan) to generate pOseIF4AIIb and pOsRH8.

To examine the subcellular distribution of OseIF4AIIb and OsRH8, full-length cDNA fragments were excised from pOseIF4AIIb and pOsRH8 using *Asc*I and *Not*I, and subsequently inserted into the pENTR-TOPO vector at the corresponding restriction sites, generating the pOseIF4AIIb-ENTR and pOsRH8-ENTR constructs. Recombination was performed using LR Clonase (Invitrogen, Carlsbad, CA, USA) to transfer the *OseIF4AIIb* or *OsRH8* DNA fragment from the entry clone to the destination vectors pMDC85 or pMDC85m, thereby resulting in OseIF4AIIb-GFP, OsRH8-GFP, OseIF4AIIb-mCherry, or OsRH8-mCherry expression constructs. Plasmids encoding OsCAF1s-GFP had been previously generated in an earlier study [23].

For the construction of the *OseIF4AIIb and OsRH8* RNAi vectors, a 291- or 342-base pair (bp) DNA fragment of the 3′ untranslated region (UTR) of *OseIF4AIIb* or *OsRH8* was amplified by PCR using gene-specific primers (Supplementary Table S1). These DNA fragments were cloned into the yT&A cloning vector to generate plasmids pOseIF4AIIbRi and pOsRH8Ri. *GFP* cDNA was amplified by PCR using forward and reverse primers (Supplementary Table S1) and then subcloned into the yT&A cloning vector to construct pGFPRi. The *OseIF4AIIb and OsRH8* RNAi DNA fragments were excised from pOseIF4AIIbRi or pOsRH8Ri by *Eco*RI and *Bam*HI digestion, while the *GFP* fragment was released from pGFPRi by *Eco*RI digestion. The *OseIF4AIIb or OsRH8* RNAi DNA fragment and *GFP* DNA fragment were ligated into the *Bam*HI site of the pAHC18 expression vector, resulting in pAHC18-OseIF4AIIb-Ri or pAHC18-OsRH8-Ri, respectively.

To construct an ectopic expression vector for *OsCAF1A*, the *OsCAF1A* cDNA fragment was excised from pOsCAF1A by using *Bam*HI and inserted into the *Bam*HI site of the pAHC18 expression vector, positioned between the maize (*Zea mays*) *Ubi* promoter and the *Nos* terminator to generate pAHC18-OsCAF1A. The pUbi-OsCAF1A construct was subsequently linearized by digestion with *Hind*III and subcloned into the pCAMBIA1301 binary vector to generate pCAMBIA1301-OsCAF1A.

To perform gene editing of the mutant, the genomic target sequence of *OsCAF1A*, a designed OsCAF1A-sgRNA, 20-bp DNA (72nd–91st nucleotides from the first ATG), was synthesized and fused downstream of the *OsU3* promoter. The sgRNA-Cas9 plasmid was generated using the Gateway LR recombination system with a binary vector sgRNA-Cas9 [51] and the *OsU3* promoter-sgRNA vector. The DNA fragment was inserted into the *Bsa*I site of the pOs-sgRNA entry vector [51], resulting in the generation of an *OsU3* promoter-OsCAF1A-sgRNA expression cassette. Using LR Clonase (Invitrogen), the OsCAF1A-sgRNA expression cassette was inserted into the sgRNA-Cas9 vector [51] via recombination to generate the OsCAF1A-sgRNA-Cas9 plasmid.

The plasmids used in the BiFC analyses were full-length *OsCAF1s*, generated previously [52]. To generate *OseIF4AIIb* and *OsRH8* fusion constructs, the pOseIF4AIIb-ENTR or pOsRH8-ENTR vectors were recombined into the binary vectors pSAT4- DEST-nEYFP-C1 and pSAT5-DEST-cEYFP-C1, respectively, using LR Clonase (Invitrogen), resulting in the *OseIF4AIIb*–YFP or *OsRH8*–YFP fusion constructs.

### 4.4. RT-PCR and RT-qPCR Analyses

Total RNA was isolated from rice suspension-cultured cells or seedlings using the TRIzol reagent (Invitrogen), following the manufacturer’s protocol. Possible genomic DNA contamination was eliminated by DNase I (NEB) treatment. RNA integrity was assessed by agarose gel electrophoresis, and purity was confirmed using a spectrophotometer (NanoDrop), ensuring A260/A280 and A260/A230 ratios were within acceptable ranges. First-strand cDNA was synthesized using ReverTra Ace (Toyobo) with oligo(dT) primers under the following conditions: 65 °C for 5 min, 4 °C for 5 min, 42 °C for 60 min, 85 °C for 20 min, and 25 °C for 10 min. The synthesized cDNA was validated by PCR using rice *Actin 1* (*OsAct1*) with 25 cycles of 95 °C for 20 s, 55 °C for 45 s, and 72 °C for 1 min. Amplification products were confirmed by agarose gel electrophoresis. A 10-fold dilution of the synthesized first-strand cDNA was used as a template for RT-qPCR analysis, performed on a PikoReal Real-Time PCR System (Thermo Fisher), according to the manufacturer’s instructions. The PCR procedure was conducted independently at least three times. The relative mRNA expression levels of the target gene were normalized using rice *Act1* mRNA, which served as an internal control. Data analysis was performed using PikoReal software (Thermo Fisher, Waltham, MA, USA) following the manufacturer’s guidelines. The gene-specific primers used for RT-qPCR are listed in Supplementary Table S1.

### 4.5. Standard Curves and Absolute Quantification

Four distinct *OsCAF1*-containing plasmids were generated and expressed into DH5α competent *Escherichia coli* cells. Following plasmid extraction, the concentration of each *OsCAF1*-containing DNA plasmid was measured and normalized. Standard curves were generated by plotting the Ct values of serially diluted DNA plasmid standards against the logarithm of their known concentrations, as previously described [53,54]. The efficiency of the PCR was assessed from the slope of the standard curves, with high efficiency indicating acceptable performance. The absolute expression levels of the target *OsCAF1* genes were quantified by interpolating the Ct values of the unknown samples against the standard curve. Each experiment was conducted with three independent biological experiments. The primers used are provided in Supplementary Table S1.

### 4.6. Plant Transformation

Immature embryos from germinated rice seeds were cultured on an N6 solid medium supplemented with 9 μM 2,4-dichlorophenoxyacetic acid (2,4-D) to induce callus formation. Rice transformation was carried out using *Agrobacterium tumefaciens* strain EHA105, following a previously established protocol [51]. Transformed calli were selected on N6 medium with 3% (*w*/*v*) sucrose and 50 mg∙L^−1^ hygromycin B, as described previously [51].

### 4.7. Phenotypic Analysis of Heat Stress-Treated Plants

To conduct a comparative analysis of seedling morphology between wild-type (WT) and *OsCAF1A* transgenic lines under different conditions, 7-day-old rice seedlings were subjected to an additional 14 days growth period under varying temperature conditions, including normal temperature (28 °C) or sublethal high-temperature conditions (32 °C and 37 °C). The growth of the seedlings was determined based on shoot length measurements.

In the heat stress treatment, 7-day-old seedlings were exposed to 42 °C in a growth chamber for 7 days, followed by a recovery period at 28 °C. Plants were considered to have survived if they exhibited green, healthy leaves and resumed growth after 14 days of recovery. A minimum of three independent biological experiments were conducted.

In the electrolyte leakage assay, 2-week-old seedlings of the WT and *OsCAF1A* transgenic lines were treated at 42 °C for 5 days and then assessed for electrolyte leakage (EL) as described previously [30]. One-centimeter leaf segments were excised from the fourth leaf of each seedling. The heat-induced electrolyte leakage (%) was calculated as follows: electrolyte leakage (heat)/total electrolyte leakage × 100. Five plants from each independent line were used, and the experiments were replicated three times.

### 4.8. Subcellular Localization Analysis and Bimolecular Fluorescence Complementation (BiFC) Assay

To confirm the subcellular localization, the coding sequences of the target genes were amplified and inserted into the pMDC85 vectors driven by the *2X35S:* promoter. The GFP-fused constructs were co-transformed into rice protoplasts with *2X35S: OsDCP2* or *2X35S: AteIF3B1* as the PB or SG marker, respectively. The *2X35S: GFP* construct was used as a control. Rice protoplast transformation was performed as previously described [30]. After transformation, fluorescence signals were captured 6–8 h post-co-transformation using an inverted fluorescence microscope (Olympus IX71, Olympus, Tokyo, Japan).

In the BiFC analysis, different combinations of expression vectors carrying YFPN- and YFPC-fused genes were co-expressed in rice protoplasts. Fluorescence signals were detected 6–8 h post-co-transformation using an inverted fluorescence microscope (Olympus IX71) fitted with an Olympus UMWIBA3 filter.

### 4.9. Co-Immunoprecipitation and Mass Spectrometry (IP-MS) Analysis

A co-immunoprecipitation (Co-IP) assay combined with mass spectrometry (IP-MS) was conducted using GFP-fusion OsCAF1A (OsCAF1A-GFP) to identify the putative interactors of OsCAF1A in rice cells, employing the GFP-trapping method as described by Yilmazer et al. [40]. Freshly harvested calli were frozen in liquid nitrogen and ground into a fine powder. Total protein extraction was performed using an appropriate lysis buffer (10 mM Tris-HCl pH 7.5, 150 mM NaCl; 0.5 mM EDTA, 0.5% NP-40, and 1 mM PMSF). The lysed supernatant was incubated with 30 μL of pre-equilibrated GFP-Trap_A beads in wash buffer (10 mM Tris/Cl pH 7.5; 150 mM NaCl; 0.5 mM EDTA) and gently rotated at 4 °C for 2 h. The beads underwent three washes with 500 µL of wash buffer to remove non-specifically bound proteins. Mass spectrometry (IP-MS) analysis was conducted following the method described by Liu et al. [41]. Peptides for liquid chromatography-tandem mass spectrometry (LC-MS/MS) were prepared through in-gel digestion with sequencing-grade modified porcine trypsin (Promega, Madison, WI, USA) at 37 °C for 16 h. LC-MS/MS analysis was performed using reverse-phase liquid chromatography (RPLC) coupled to an Orbitrap Elite ETD mass spectrometer (Thermo Fisher, San Jose, CA, USA) to achieve high-resolution analysis. The chromatographic separation was conducted using a Zorbax 300SB-C18 column (0.3 × 5 mm; Agilent Technologies, Wilmington, DE, USA) for initial peptide trapping, followed by analytical separation on a HydroRP column (2.5 μm, 75 μm I.D. × 20 cm, 15-μm tip, homemade). The mobile phase consisted of Buffer A (0.1% formic acid in water) and Buffer B (99.9% acetonitrile/0.1% formic acid). A multi-step gradient elution over 70 min was applied at a flow rate of 0.3 μL/min, optimized for nano-electrospray ionization (nano-ESI). The mass spectrometry analysis was conducted in data-dependent acquisition (DDA) mode, selecting the 20 most abundant precursor ions for MS/MS fragmentation. The MS scan range was 400–2000 Da, with a resolution of 120,000 at *m*/*z* 400. A 40 s dynamic exclusion window (15 ppm tolerance) was applied to minimize repeated precursor selection. Electrospray ionization (ESI) parameters were set at 2.0 kV spray voltage and 200 °C capillary temperature. Automatic Gain Control (AGC) was set to 3 × 10^6^ ions for full scans and 3 × 10^3^ ions for MS/MS scans. Data analysis was conducted using Proteome Discoverer software (version 1.4, Thermo Fisher Scientific). The MS/MS spectra were searched against the UniProt and NCBI (RefSeq) databases using the Mascot search engine (Matrix Science, London, UK; version 2.5) for protein identification.

### 4.10. Statistical Analysis

Statistical analyses were conducted using Student’s *t*-test, with * *p* < 0.05 considered the threshold for a significant difference. All statistical analyses were performed using Microsoft Excel.

## 5. Conclusions

So far, the subcellular localization of proteins has been documented in both developmental processes and responses to biotic and abiotic stresses in plants. However, most of the physical functions and subcellular localization of rice OsCAF1 proteins remain uncharacterized. This study investigated OsCAF1A function and localization in rice under heat stress. Expression analysis of *OsCAF1* genes indicated that *OsCAF1A* was highly expressed compared to the other *OsCAF1* genes in rice seedlings, and its expression was further upregulated in response to elevated temperatures. Gain- and loss-of-function analyses revealed that *OsCAF1A* overexpression conferred heat stress tolerance in transgenic rice seedlings, whereas its mutation increased susceptibility to heat stress. Except for OsCAF1B, other OsCAF1s are localized in the cytoplasm and are predominantly recruited to PBs under elevated temperature conditions. Co-immunoprecipitation (co-IP) indicated that OseIF4AIIb and OsRH8, two DEAD-box RNA helicase proteins located in the cytoplasm and PBs, respectively, were partners of OsCAF1. We now show that these DEAD-box RNA helicase proteins have distinct and opposing effects on the re-localization of OsCAF1A. OseIF4AIIb inhibited the re-localization of OsCAF1A to PBs, whilst OsRH8 enhanced the recruitment of OsCAF1A to PBs. These findings demonstrate the crucial role of OsCAF1A in heat-stress tolerance and its regulated re-localization in response to heat stress in rice.

## Figures and Tables

**Table 1:** List of the components of the OsCCR4-NOT complex identified by IP-MS analysis.

Accession of Gene	Gene Name	MW [kDa]	Normalized PSMs		Gene Description
			OsCAF1A -GFP	GFP Only	
XP_015614670.1	*OsCCR4a*	67.2	65.1	-	Carbon catabolite repressor protein 4 homolog 1
XP_025879986.1	*OsCCR4b*	67.5	44.0	-	Carbon catabolite repressor protein 4 homolog 1
XP_015614203.1	*OsNOT1*	267.6	118.2	-	CCR4-NOT transcription complex subunit 1 isoform X1
XP_015627126.1	*OsNOT2a*	66.3	53.2	-	Probable NOT transcription complex subunit VIP2
XP_015630454.1	*OsNOT2*	60.9	63.2	-	Probable NOT transcription complex subunit VIP2 isoform X3
XP_015630453.1	*OsNOT2b*	66.5	63.0	-	NOT2/NOT3/NOT5 family protein
XP_015629837.1	*OsNOT9c*	35.6	30.2	-	CCR4-NOT transcription complex subunit 9
XP_025883045.1	*OsNOT9*	35.2	19.2	-	CCR4-NOT transcription complex subunit 9 isoform X2

**Table 2:** List of potential interactor proteins with the highest number of normalized PSMs detected by IP-MS analysis.

Accession of Gene	Accession Number of Proteins	MW [kDa]	Normalized PSMs		Gene Description
			OsCAF1A -GFP	GFP Only	
XP_015626460.1	Q6Z2Z4	47.1	62.3	-	Eukaryotic initiation factor 4AIIb
XP_015627069.1	Q6H7S	58.1	39.4	-	DEAD-box ATP-dependent RNA helicase 8-like
XP_015650041.1	P41095	34.4	34.8	-	60S acidic ribosomal protein P0-like
XP_015632031.1	Q10RW9	61.0	33.0	-	Chaperonin CPN60-1, mitochondrial
XP_015614255.1	Q7XE16	89.8	29.3	-	Cell division control protein 48 homolog E
XP_015632250.1	C7IZI5	44.5	24.7	-	60S ribosomal protein L4
XP_015630377.1	A3AIN7	28.0	22.9	-	40S ribosomal protein S6
XP_025881412.1	B7F8T1	27.3	21.0	-	Uncharacterized protein LOC9269814

## Data Availability

All data supporting the findings of this study are included in the main text, and Supplementary Materials available online. Additional details can be from the corresponding author.

## Supplementary Materials

The following supporting information can be downloaded at: https://www.mdpi.com/article/10.3390/plants14071036/s1, Figure S1. High-temperature-induced re-localization of OsCAF1-GFP to cytoplasmic foci in transgenic plants. Figure S2. Characterization of OsCAF1A-GFP transgenic line. Figure S3. Interaction between OsCAF1A and OseIF4AIIb or OsRH8 proteins using BiFC assays in onion epidermal cells. Figure S4. Gene expression patterns of *OseIF4AIIb* and *OsRH8*. Figure S5. OsIF4AIIb suppressed the re-localization of OsCAF1H to cytoplasmic foci, whereas OsRH8 promoted this process. Figure S6. Standard curve for absolute quantitative RT-PCR of four different *OsCAF1* genes. Figure S7. Characterization of *OsCAF1A* mutant and overexpression lines. Table S1. Primers used in this study.

## Abbreviations

BiFC	Bimolecular fluorescence complementation
CCR4-NOT	Carbon Catabolite Repression 4-Negative On TATA-less
CHX	Cycloheximide
co-IP	co-immunoprecipitation
CRISPR/Cas9	Clustered regularly interspaced palindromic repeats
DMSO	Dimethyl sulfoxide
LC-MS/MS	Liquid chromatography-tandem mass spectrometry
PB	Processing body
SG	Stress granule
TNG67	Tainung 67
