# Supplementary material for: Characterization of OsCAF1 Protein Function in Rice Response to Thermal Stress
Source: Plants (Basel). 2025 Mar 27;14(7):1036. doi: 10.3390/plants14071036 (PMC11990703; doi:10.3390/plants14071036)
Supplement: Supplementary file 1 [file plants-14-01036-s001.zip › plants-3535578-supplementary-Figure S1-7.pptx]

## Slide 1
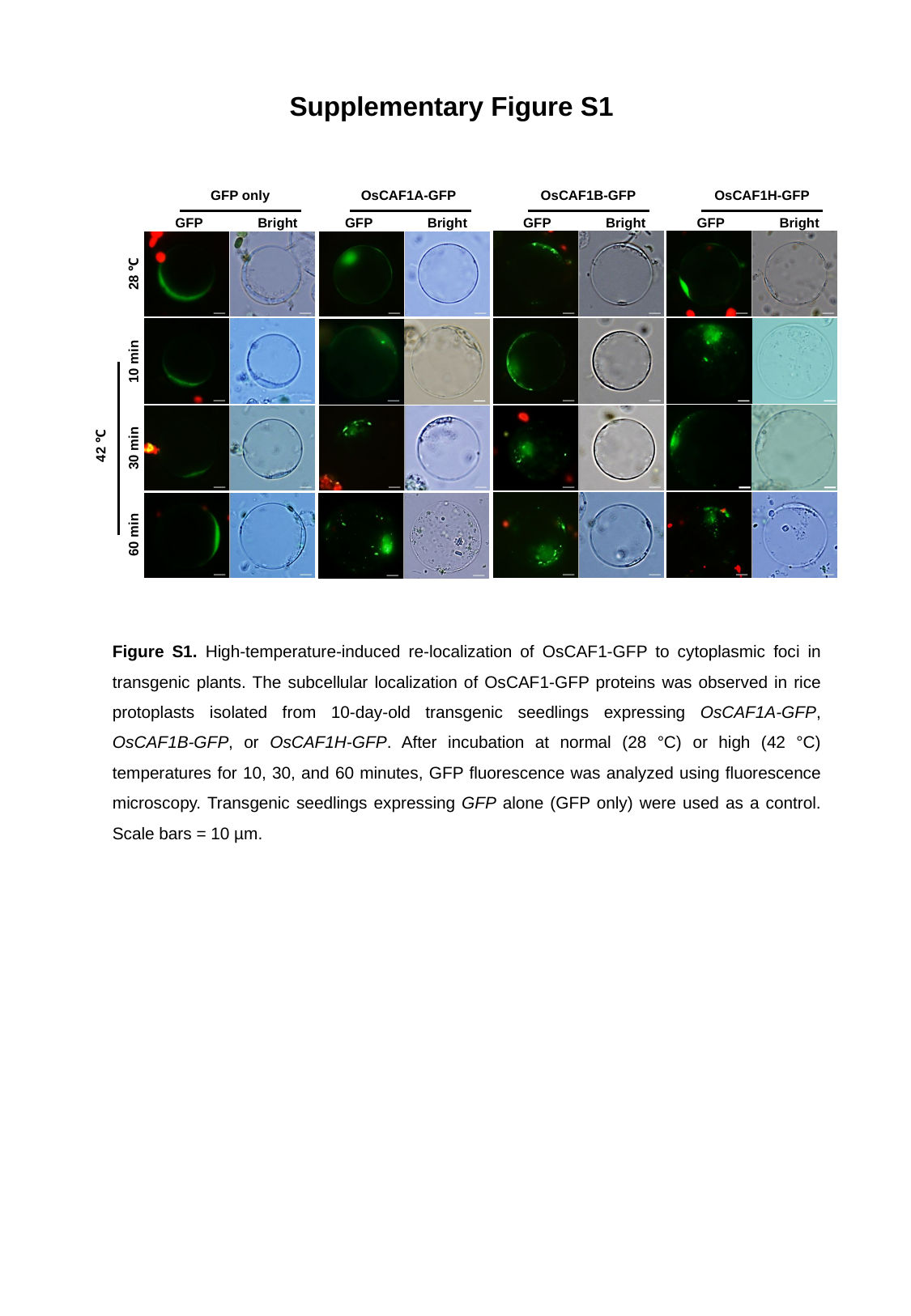

Supplementary Figure S1
OsCAF1H-GFP
OsCAF1A-GFP
OsCAF1B-GFP
GFP only
GFP
Bright
GFP
Bright
GFP
Bright
GFP
Bright
28 ℃
10 min
42 ℃
30 min
60 min
Figure S1. High-temperature-induced re-localization of OsCAF1-GFP to cytoplasmic foci in transgenic plants. The subcellular localization of OsCAF1-GFP proteins was observed in rice protoplasts isolated from 10-day-old transgenic seedlings expressing OsCAF1A-GFP, OsCAF1B-GFP, or OsCAF1H-GFP. After incubation at normal (28 °C) or high (42 °C) temperatures for 10, 30, and 60 minutes, GFP fluorescence was analyzed using fluorescence microscopy. Transgenic seedlings expressing GFP alone (GFP only) were used as a control. Scale bars = 10 µm.

## Slide 2
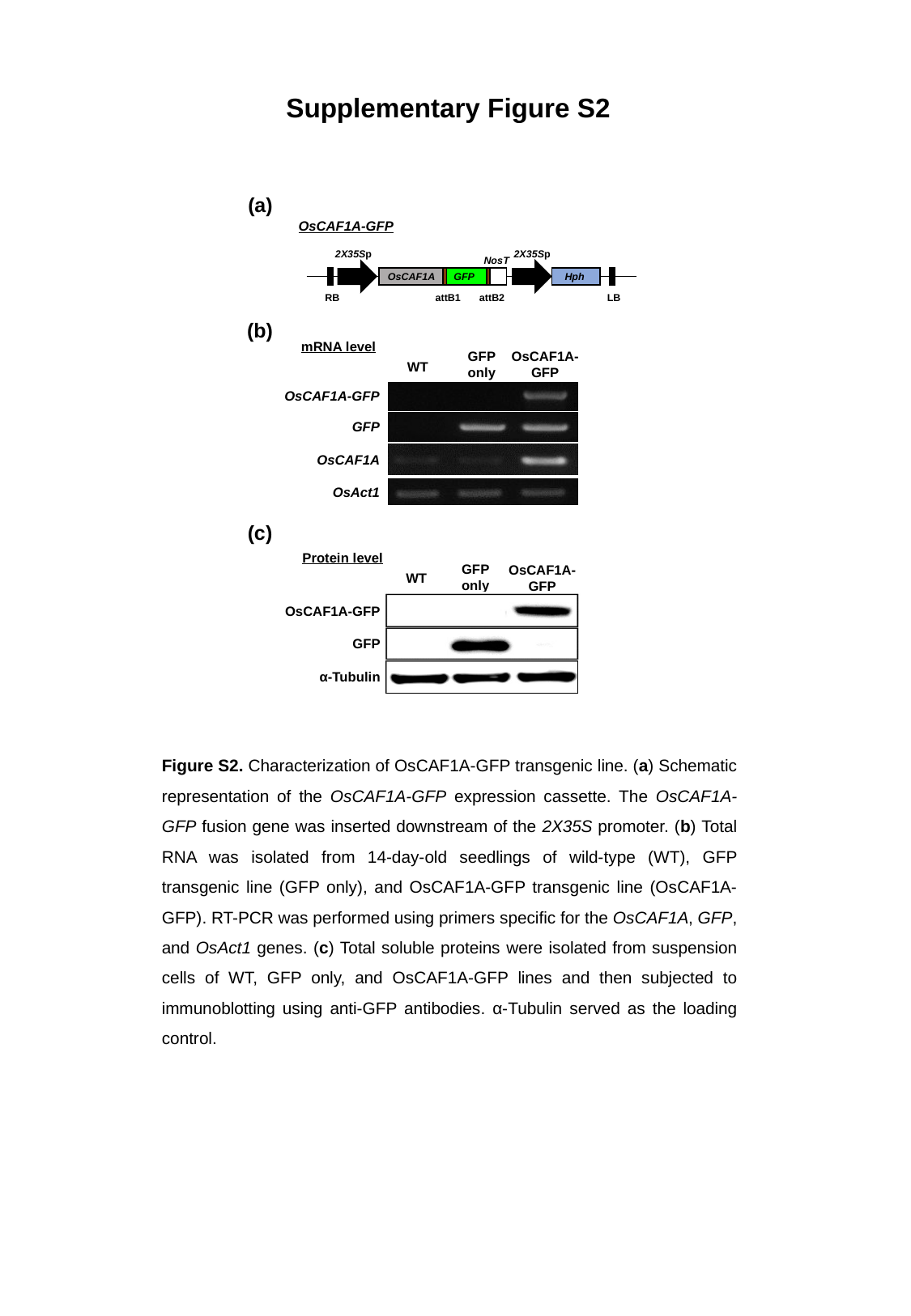

Supplementary Figure S2
(a)
OsCAF1A-GFP
2X35Sp
2X35Sp
NosT
OsCAF1A
GFP
Hph
RB
attB1
attB2
LB
(b)
mRNA level
GFP
only
OsCAF1A-GFP
WT
OsCAF1A-GFP
GFP
OsCAF1A
OsAct1
(c)
Protein level
GFP
only
OsCAF1A-GFP
WT
OsCAF1A-GFP
GFP
α-Tubulin
Figure S2. Characterization of OsCAF1A-GFP transgenic line. (a) Schematic representation of the OsCAF1A-GFP expression cassette. The OsCAF1A-GFP fusion gene was inserted downstream of the 2X35S promoter. (b) Total RNA was isolated from 14-day-old seedlings of wild-type (WT), GFP transgenic line (GFP only), and OsCAF1A-GFP transgenic line (OsCAF1A-GFP). RT-PCR was performed using primers specific for the OsCAF1A, GFP, and OsAct1 genes. (c) Total soluble proteins were isolated from suspension cells of WT, GFP only, and OsCAF1A-GFP lines and then subjected to immunoblotting using anti-GFP antibodies. α-Tubulin served as the loading control.

## Slide 3
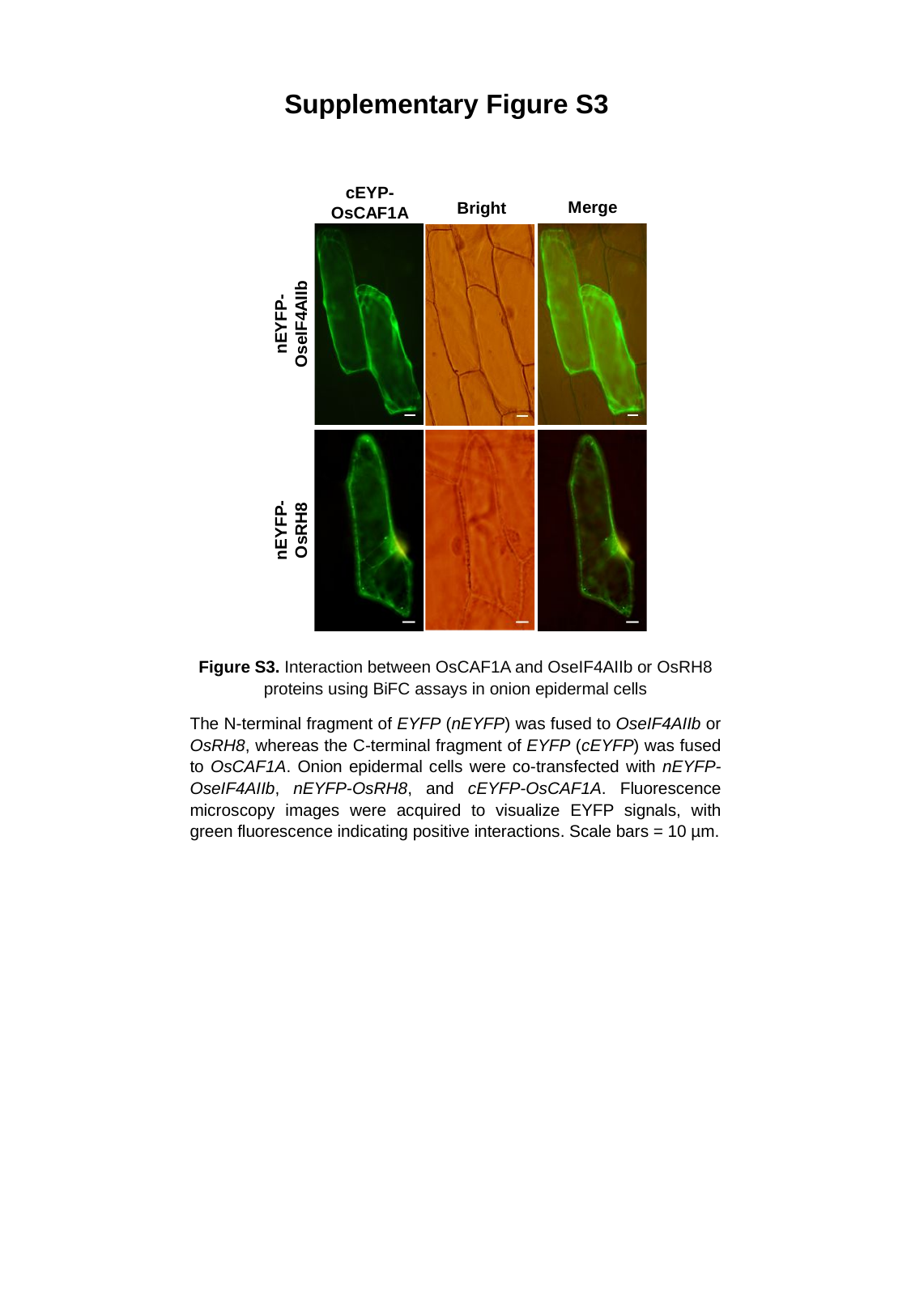

Supplementary Figure S3
Figure S3. Interaction between OsCAF1A and OseIF4AIIb or OsRH8 proteins using BiFC assays in onion epidermal cells
The N-terminal fragment of EYFP (nEYFP) was fused to OseIF4AIIb or OsRH8, whereas the C-terminal fragment of EYFP (cEYFP) was fused to OsCAF1A. Onion epidermal cells were co-transfected with nEYFP-OseIF4AIIb, nEYFP-OsRH8, and cEYFP-OsCAF1A. Fluorescence microscopy images were acquired to visualize EYFP signals, with green fluorescence indicating positive interactions. Scale bars = 10 µm.

## Slide 4
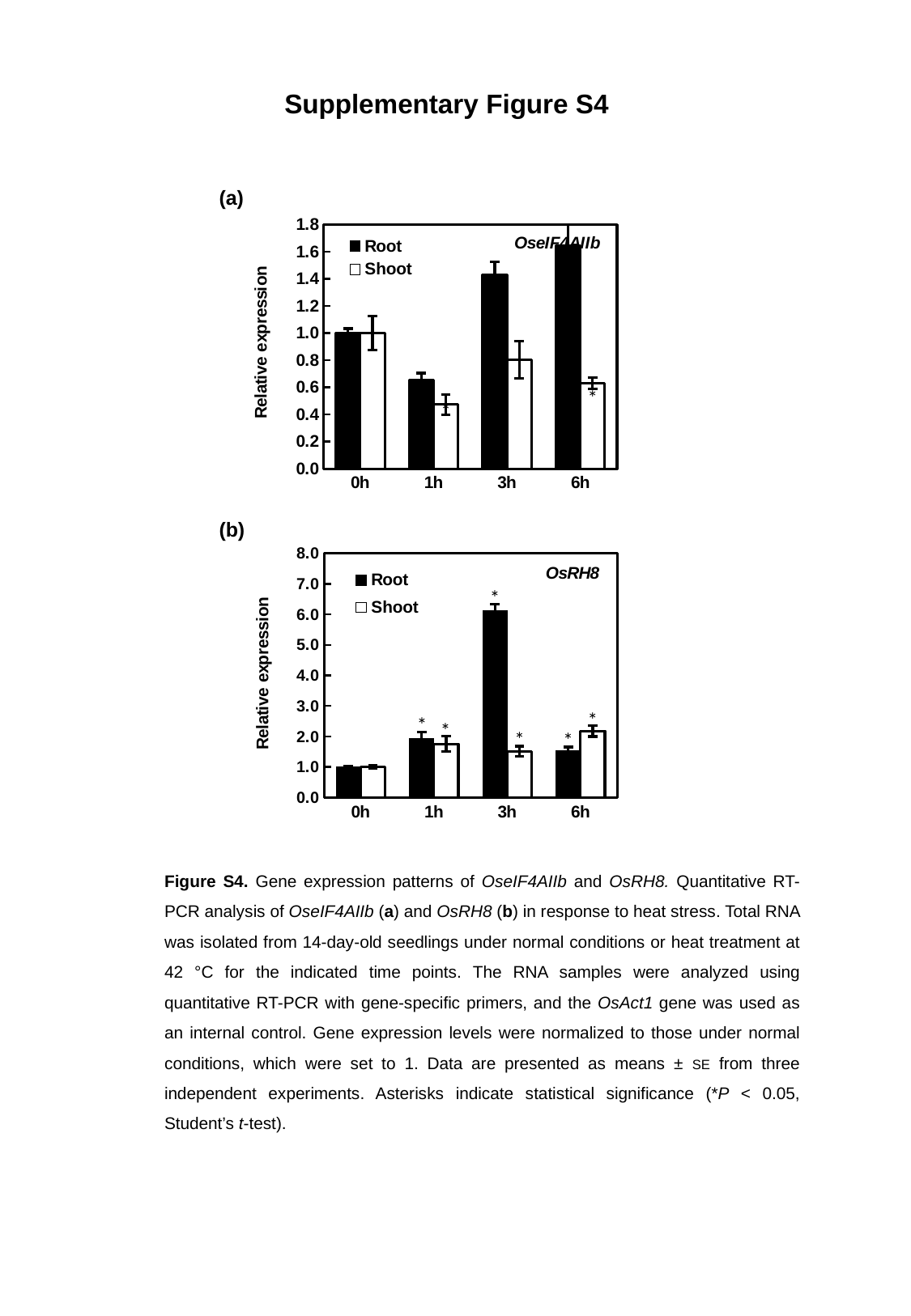

Supplementary Figure S4
(a)
### Chart: OseIF4AIIb
| Category | Root | Shoot |
|---|---|---|
| 0h | 1.0 | 1.0 |
| 1h | 0.6552113076638892 | 0.4723381537357965 |
| 3h | 1.4312593159395215 | 0.8028482914983777 |
| 6h | 1.6430005201546811 | 0.6292011510042183 |(b)
### Chart: OsRH8
| Category | Root | Shoot |
|---|---|---|
| 0h | 1.0 | 1.0 |
| 1h | 1.929970201251183 | 1.7548648359522148 |
| 3h | 6.119976418888467 | 1.5090665427784196 |
| 6h | 1.524867668419497 | 2.1818297601343204 |*
*
*
*
*
*
*
*
*
*
*
Figure S4. Gene expression patterns of OseIF4AIIb and OsRH8. Quantitative RT-PCR analysis of OseIF4AIIb (a) and OsRH8 (b) in response to heat stress. Total RNA was isolated from 14-day-old seedlings under normal conditions or heat treatment at 42 °C for the indicated time points. The RNA samples were analyzed using quantitative RT-PCR with gene-specific primers, and the OsAct1 gene was used as an internal control. Gene expression levels were normalized to those under normal conditions, which were set to 1. Data are presented as means ± SE from three independent experiments. Asterisks indicate statistical significance (*P < 0.05, Student’s t-test).

## Slide 5
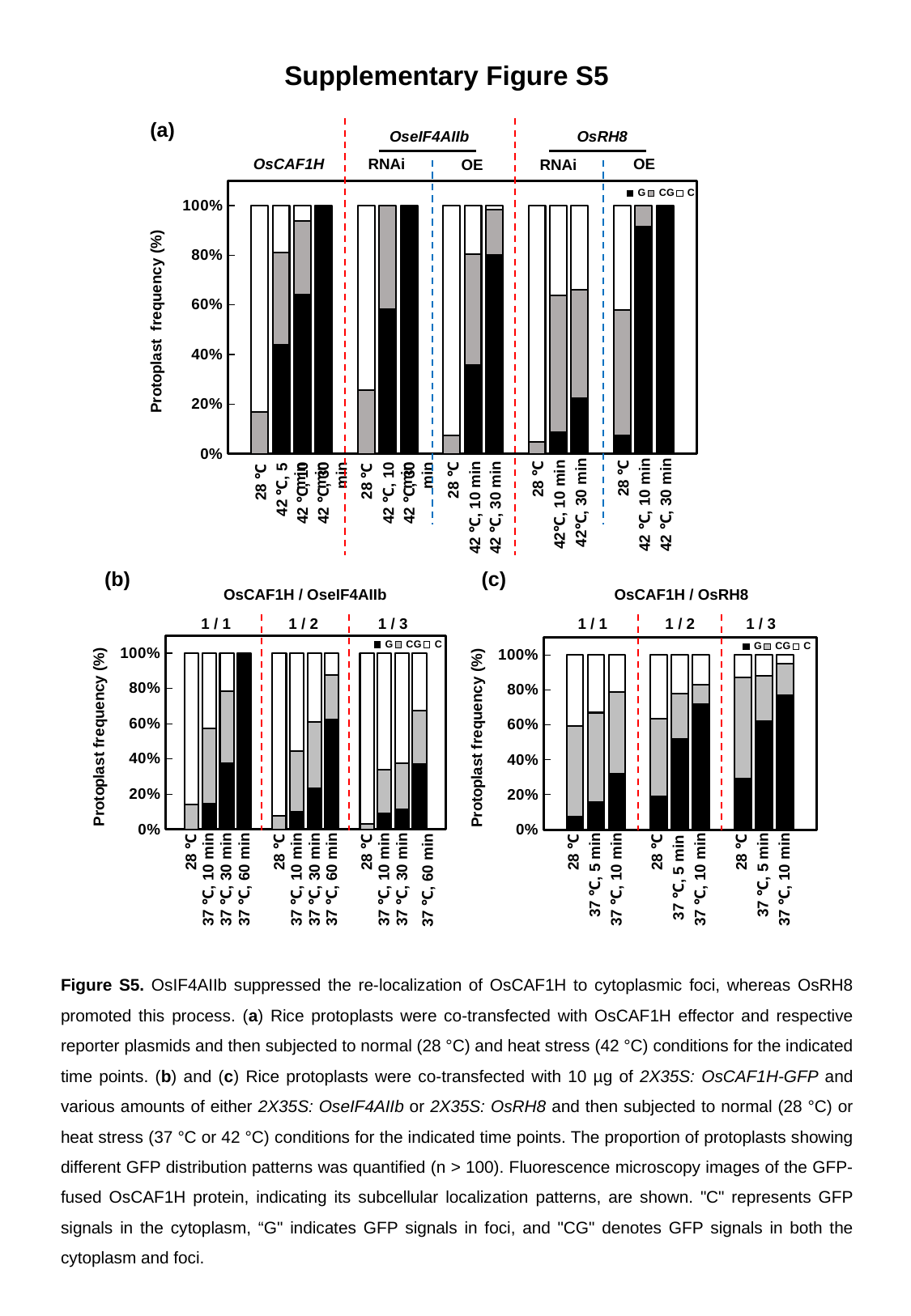

Supplementary Figure S5
(a)
OsRH8
OseIF4AIIb
OE
RNAi
RNAi
OE
OsCAF1H
### Chart
| Category | G | CG | C |
|---|---|---|---|
| | None | None | None |
| 28 °C | 0.0 | 0.16877687529861443 | 0.8312231247013856 |
| 42 °C, 5min | 0.44045911047345765 | 0.3703172325840905 | 0.18922365694245177 |
| 42 °C, 10min | 0.6416416416416416 | 0.2977620477620478 | 0.0605963105963106 |
| 42 °C, 30min | 1.0 | 0.0 | 0.0 |
| | None | None | None |
| 28 °C | 0.0 | 0.2569371879716707 | 0.7430628120283292 |
| 42 °C, 10min | 0.5836027771511643 | 0.41639722284883574 | 0.0 |
| 42 °C, 30min | 1.0 | 0.0 | 0.0 |
| | None | None | None |
| 28 °C | 0.0 | 0.07323232323232323 | 0.9267676767676768 |
| 42 °C, 10min | 0.3573426126617616 | 0.4473884686650644 | 0.19526891867317397 |
| 42 °C, 30min | 0.7998712998712998 | 0.18318318318318316 | 0.016945516945516947 |
| | None | None | None |
| 28 °C | 0.0 | 0.04884792626728111 | 0.9511520737327189 |
| 42 °C, 10min | 0.088327721661055 | 0.5503367003367003 | 0.3613355780022447 |
| 42 °C, 30min | 0.2251984126984127 | 0.43452380952380953 | 0.34027777777777773 |
| | None | None | None |
| 28 °C | 0.07380952380952381 | 0.5055555555555556 | 0.42063492063492064 |
| 42 °C, 10min | 0.9135375494071146 | 0.08646245059288538 | 0.0 |
| 42 °C, 30min | 1.0 | 0.0 | 0.0 |28 ℃
28 ℃
28 ℃
28 ℃
28 ℃
42 ℃, 5 min
42℃, 30 min
42℃, 10 min
42 ℃, 10 min
42 ℃, 30 min
42 ℃, 10 min
42 ℃, 30 min
42 ℃, 10 min
42 ℃, 10 min
42 ℃, 30 min
42 ℃, 30 min
(b)
OsCAF1H / OseIF4AIIb
### Chart
| Category | G | CG | C |
|---|---|---|---|
| | None | None | None |
| 1H-GFP + 1.IIB+mcherry | 0.0 | 0.14251207729468598 | 0.8574879227053139 |
| | 0.14474308300395256 | 0.42779973649538866 | 0.4274571805006588 |
| | 0.37672811059907835 | 0.4062980030721966 | 0.21697388632872502 |
| | 1.0 | 0.0 | 0.0 |
| | None | None | None |
| 1H-GFP + 2.IIB+mcherry | 0.0 | 0.07647907647907648 | 0.9235209235209235 |
| | 0.09999999999999999 | 0.3419753086419753 | 0.5580246913580247 |
| | 0.23174603174603173 | 0.37976190476190474 | 0.38849206349206344 |
| | 0.6235309017223911 | 0.2541540020263424 | 0.12231509625126645 |
| | None | None | None |
| 1H-GFP + 3.IIB+mcherry | 0.0 | 0.031746031746031744 | 0.9682539682539683 |1 / 3
1 / 2
1 / 1
28 ℃
28 ℃
28 ℃
37 ℃, 10 min
37 ℃, 60 min
37 ℃, 30 min
37 ℃, 10 min
37 ℃, 30 min
37 ℃, 10 min
37 ℃, 30 min
37 ℃, 60 min
(c)
OsCAF1H / OsRH8
### Chart
| Category | G | CG | C |
|---|---|---|---|
| | None | None | None |
| 1H-GFP + 1.RH8-mcherry | 0.07380952380952381 | 0.52 | 0.41 |
| | 0.16 | 0.51 | 0.33 |
| | 0.32 | 0.47 | 0.21 |
| | None | None | None |
| 1H-GFP + 2.RH8-mcherry | 0.21 | 0.49 | 0.4 |
| | 0.52 | 0.26 | 0.22 |
| | 0.72 | 0.11 | 0.17 |
| | None | None | None |
| 1H-GFP + 3.RH8-mcherry | 0.29 | 0.58 | 0.13 |1 / 3
1 / 2
1 / 1
28 ℃
28 ℃
28 ℃
37 ℃, 5 min
37 ℃, 5 min
37 ℃, 5 min
37 ℃, 10 min
37 ℃, 10 min
37 ℃, 10 min
37 ℃, 60 min
Figure S5. OsIF4AIIb suppressed the re-localization of OsCAF1H to cytoplasmic foci, whereas OsRH8 promoted this process. (a) Rice protoplasts were co-transfected with OsCAF1H effector and respective reporter plasmids and then subjected to normal (28 °C) and heat stress (42 °C) conditions for the indicated time points. (b) and (c) Rice protoplasts were co-transfected with 10 µg of 2X35S: OsCAF1H-GFP and various amounts of either 2X35S: OseIF4AIIb or 2X35S: OsRH8 and then subjected to normal (28 °C) or heat stress (37 °C or 42 °C) conditions for the indicated time points. The proportion of protoplasts showing different GFP distribution patterns was quantified (n > 100). Fluorescence microscopy images of the GFP-fused OsCAF1H protein, indicating its subcellular localization patterns, are shown. "C" represents GFP signals in the cytoplasm, “G" indicates GFP signals in foci, and "CG" denotes GFP signals in both the cytoplasm and foci.

## Slide 6
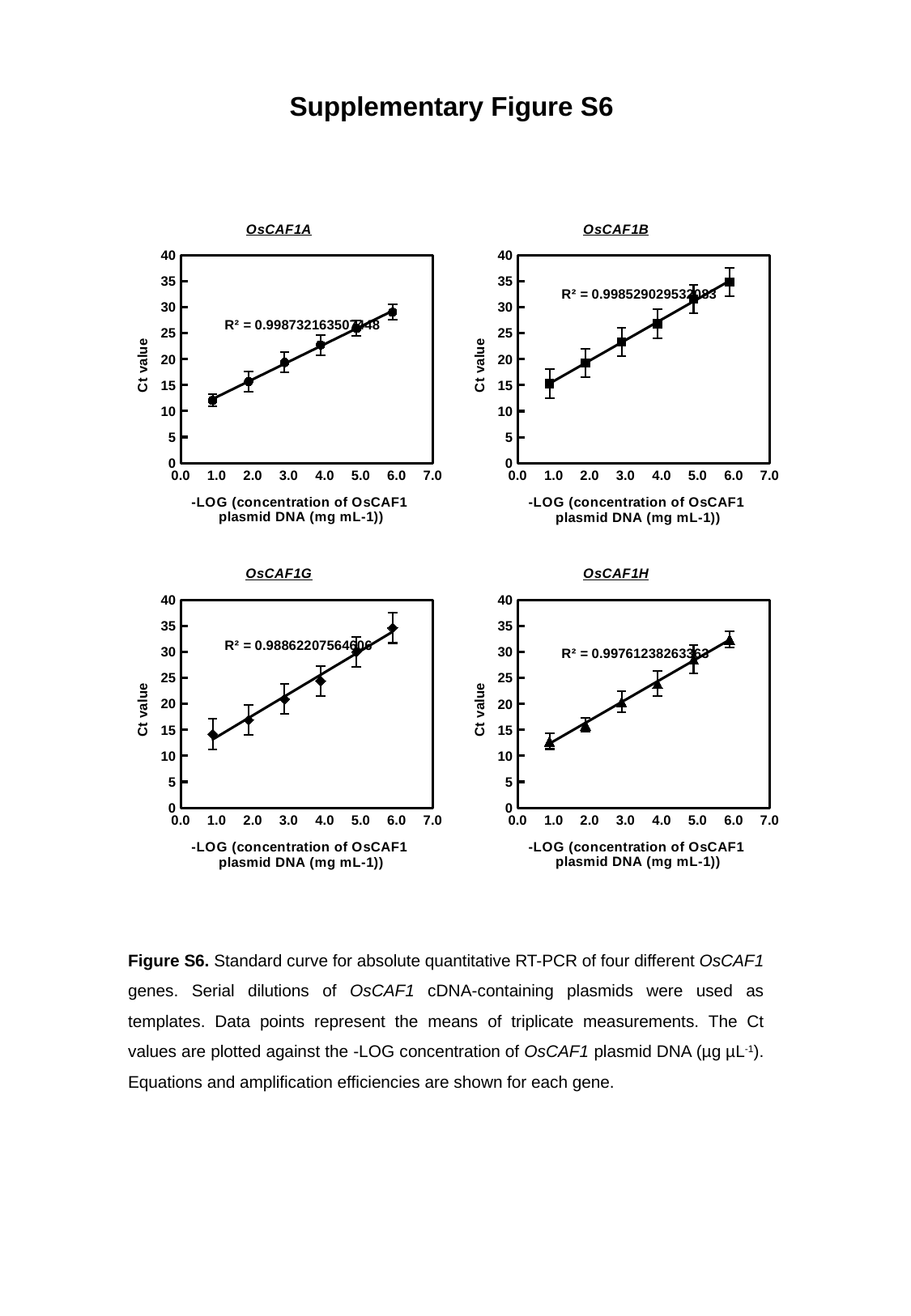

Supplementary Figure S6
### Chart: OsCAF1B
| Category | OsCAF1B |
|---|---|
### Chart:
| Category | OsCAF1A |
|---|---|
### Chart:
| Category | OsCAF1G |
|---|---|
### Chart:
| Category | OsCAF1H |
|---|---|Figure S6. Standard curve for absolute quantitative RT-PCR of four different OsCAF1 genes. Serial dilutions of OsCAF1 cDNA-containing plasmids were used as templates. Data points represent the means of triplicate measurements. The Ct values are plotted against the -LOG concentration of OsCAF1 plasmid DNA (µg µL-1). Equations and amplification efficiencies are shown for each gene.

## Slide 7
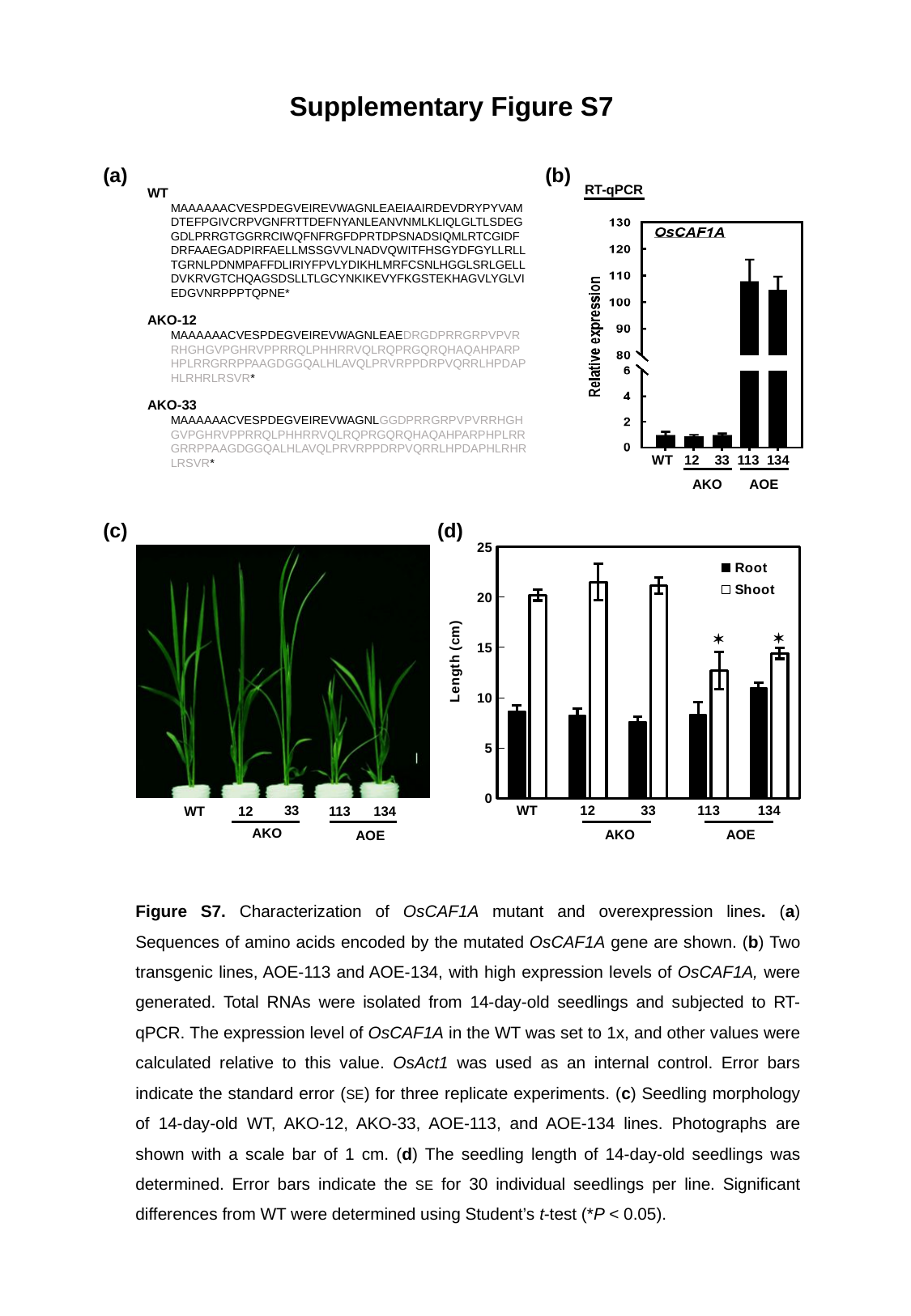

Supplementary Figure S7
(b)
RT-qPCR
WT 12 33 113 134
AKO
AOE
(a)
WT
MAAAAAACVESPDEGVEIREVWAGNLEAEIAAIRDEVDRYPYVAMDTEFPGIVCRPVGNFRTTDEFNYANLEANVNMLKLIQLGLTLSDEGGDLPRRGTGGRRCIWQFNFRGFDPRTDPSNADSIQMLRTCGIDFDRFAAEGADPIRFAELLMSSGVVLNADVQWITFHSGYDFGYLLRLLTGRNLPDNMPAFFDLIRIYFPVLYDIKHLMRFCSNLHGGLSRLGELLDVKRVGTCHQAGSDSLLTLGCYNKIKEVYFKGSTEKHAGVLYGLVIEDGVNRPPPTQPNE*
AKO-12
MAAAAAACVESPDEGVEIREVWAGNLEAEDRGDPRRGRPVPVRRHGHGVPGHRVPPRRQLPHHRRVQLRQPRGQRQHAQAHPARPHPLRRGRRPPAAGDGGQALHLAVQLPRVRPPDRPVQRRLHPDAPHLRHRLRSVR*
AKO-33
MAAAAAACVESPDEGVEIREVWAGNLGGDPRRGRPVPVRRHGHGVPGHRVPPRRQLPHHRRVQLRQPRGQRQHAQAHPARPHPLRRGRRPPAAGDGGQALHLAVQLPRVRPPDRPVQRRLHPDAPHLRHRLRSVR*
(d)
### Chart
| Category | Root | Shoot |
|---|---|---|
| WT | 8.666666666666666 | 20.2 |
| 12 | 8.266666666666667 | 21.5 |
| 33 | 7.566666666666666 | 21.133333333333333 |
| 113 | 8.333333333333334 | 12.700000000000001 |
| 134 | 11.0 | 14.4 |

AOE
AKO
(c)
33
WT
12
113
134
AKO
AOE
Figure S7. Characterization of OsCAF1A mutant and overexpression lines. (a) Sequences of amino acids encoded by the mutated OsCAF1A gene are shown. (b) Two transgenic lines, AOE-113 and AOE-134, with high expression levels of OsCAF1A, were generated. Total RNAs were isolated from 14-day-old seedlings and subjected to RT-qPCR. The expression level of OsCAF1A in the WT was set to 1x, and other values were calculated relative to this value. OsAct1 was used as an internal control. Error bars indicate the standard error (SE) for three replicate experiments. (c) Seedling morphology of 14-day-old WT, AKO-12, AKO-33, AOE-113, and AOE-134 lines. Photographs are shown with a scale bar of 1 cm. (d) The seedling length of 14-day-old seedlings was determined. Error bars indicate the SE for 30 individual seedlings per line. Significant differences from WT were determined using Student’s t-test (*P < 0.05).
