# Supplementary material for: Characterization of OsCAF1 Protein Function in Rice Response to Thermal Stress
Source: Plants (Basel). 2025 Mar 27;14(7):1036. doi: 10.3390/plants14071036 (PMC11990703; doi:10.3390/plants14071036)
Supplement: Supplementary file 1 [file plants-14-01036-s001.zip › plants-3535578-supplementary-Table S1.pdf]

Table S1: Primers used in this study

Primers	Sequence	Purpose(s)
OseIF4AII_NotI_F	AATGCGGCCGCCATGGCAGGAATGGCAC	OseIF4AII fusion GFP or mCherry, and BiFC
OseIF4AII_AscI_R	GGCGCGCCCTAGAGAAGGTCAGCGACGT	
OsRH8_NotI_F	AATGCGGCCGCCATGGATCCGCGCGCCAG	OsRH8 fusion GFP or mCherry, and BiFC
OsRH8_AscI_R	GGCGCGCCTTGGCAATATATAGCTTGGTCA	
OsCAF1A_NotI_F	GCGGCCGCCATGGCCGCCGCCGCCGCA	OsCAF1A fusion GFP, and BiFC
OsCAF1A_AscI_R	GGCGCGCCGGTTCATTGGGCTGAGTTGG	
OsCAF1B_NotI_F	GCGGCCGCCATGCCGTCAGAGTTCGTC	OsCAF1B fusion GFP, and BiFC
OsCAF1B_AscI_R	GGCGCGCCGGTCTCGCTGCCACCAGCGG	
OsCAF1G_NotI_F	GCGGCCGCAATGGCGACGCCGGCCGCC	OsCAF1G fusion GFP, and BiFC
OsCAF1G_AscI_R	GGCGCGCCGGGTGAGCAGCAGAGGAGGT	
OsCAF1H_NotI_F	GCGGCCGCCATGTCGGATCCCACGGCG	OsCAF1H fusion GFP, and BiFC
OsCAF1H_AscI_R	GGCGCGCCGGATGCGCACTGACAACATT	
OseIF4AII_EcoRI_F	GAATTCGTCAATGTGGAGAAGG	Construction knockdown (RNAi) of *OseIF4AII*
OseIF4AII_BamHI_R	GGATCCTAGTTGATGACAAGGGA	
OsRH8_EcoRI_F	GAATTCGACAGAGTTCCAGCGTGTATTCAC	Construction knockdown (RNAi) of *OsRH8*, qRT-PCR for *OsRH8*
OsRH8_BamHI_R	GGATCCCTAACAGGGTTAAAATGTCCTGTGAG	
OsGFP_EcoRI_F	GAATTCTGGTGAGCAAGGGCGA	Construction knockdown (RNAi), RT-PCR
OsGFP_EcoRI_R	GAATTCTTACTTGTACAGCTCGTCC	
OsCAF1A_Cas9_F	GGCAGAACCTGGAGGCGGAGATCG	Construction knockout of *OsCAF1A*
OsCAF1A_Cas9_R	AAACCGATCTCCGCCTCCAGGTTC	
OsCAF1A-qPCR-F	CCTCATCAGAATCTACTTC	qRT-PCR for *OsCAF1A*
OsCAF1A-qPCR-R	CGATCCTTTGAAGTACAC	
OsCAF1B-qPCR-F	CTCTACTTCCTCAAGCATGG	qRT-PCR for *OsCAF1B*
OsCAF1B-qPCR-F	CTCTACTTCCTCAAGCATGG	
OsCAF1G-qPCR-F	GCTTGCCGAGCTGCTTGA	qRT-PCR for *OsCAF1G*
OsCAF1G-qPCR-R	GTGAGCAGCAGACGAGGT	
OsCAF1H-qPCR-F	CCCACCGTGTATGACATCAA	qRT-PCR for O*sCAF1H*
OsCAF1H-qPCR-R	CAATGCGCACTGACAACATT	
OseIF4AIIb-qPCR-F	GCGTCTTAGGAAGTAAGGTTCTGG	qRT-PCR for *OseIF4AIIb*
OseIF4AIIb-qPCR-R	CCTTGGAATAATAACACAAGG	
Act1-F	CTGATGGACAGGTTATCACC	RT-PCR or qRT-PCR for *Act1*
Act1-R	CAGGTAGCAATAGGTATTACAG	
